# Recurrent costs in primary health care in Ethiopia: facility and disease specific unit costs and their components in government primary hospitals and health centers

**DOI:** 10.1186/s12913-020-05218-1

**Published:** 2020-05-07

**Authors:** Anubhav Agarwal, Carlyn Mann, Engida Abdella, Workie Mitiku, Abebe Alebachew, Peter Berman

**Affiliations:** 1 Global Health and Population Department, Harvard T.H. Chan School of Public Health, 677 Huntington Ave, Boston, MA 02115 USA; 2 Present address: School of Epidemiology and Public Health, University of Ottawa, 600 Peter Morand Crescent, Ottawa, K1G 5Z3 ON Canada; 3 Present Address: 500 D St SW, Washington DC, 20024 USA; 4 Breakthrough International Consultancy, PLC, Alem Birhan Plaza 4th Floor, Room No. 401 Kirkos Sub City, Kebele 17/18, House Number 005 (Near St. Urael Church), Addis Ababa, Ethiopia; 5 Present address: School of Population and Public Health, University of British Columbia, Vancouver, BC Canada

**Keywords:** Unit costs, Disease-specific costs, Health care services, Public facilities, Ethiopia

## Abstract

**Background:**

Continued investment, especially from domestic financing, is needed for Ethiopia to achieve universal health coverage and a sustainable health system over time. Understanding costs of providing health services will assist the government to mobilize adequate resources for health, and to understand future costs of changes in quality of care, service provision scope, and potential decline in external resources. This study assessed costs per unit of service output, “unit costs”, for government primary hospitals and health centers, and disease-specific services within each facility.

**Methods:**

Quantitative and qualitative data were collected from 25 primary hospitals and 47 health centers across eight of the eleven regions of Ethiopia for 2013/14, and 2014/15 and 2015/16 but only for primary hospitals, and supplemented by other related health and financial institutions records. A top-down costing approach was used to estimate unit costs for each facility by department – inpatient, outpatient, maternal and child health, and delivery. A mixed-method approach was used for the disease-specific unit costs exempt from fees.

**Results:**

Health center median unit cost was 146 Ethiopian birr (ETB) (17 PPP$, 2012), the Delivery department had the highest median unit cost (647 ETB; 76 PPP$, 2012) and Outpatient department (OPD) had the lowest (124 ETB; 14 PPP$, 2012). Primary hospital median unit cost was 339 ETB (40 PPP$, 2012), with Inpatient department having the highest median unit cost (1288 ETB; 151 PPP$, 2012), while OPD was the lowest (252 ETB; 29 PPP$, 2012). Drugs and pharmaceutical supplies accounted for most of the costs for both facilities. Among the exempted services offered, tuberculosis and antiretroviral treatment are the costliest with median unit costs from 1091 to 1536 ETB (128–180 PPP$, 2012), with drugs and supplies accounting for almost 90% of the costs.

**Conclusions:**

High unit costs of service provision could be indicative of underutilization of the primary health care system, coupled with inefficiencies associated with organization and delivery of health services. Data from this study are being used to assess efficiency and productivity among primary care facilities, facilitate premium setting for health insurance, and improve budgeting and allocating health resources for a more sustainable and effective primary health care system.

## Background

Ethiopia has made great strides in improving the health of its population over the last decade. The maternal mortality ratio dropped to 412 maternal deaths per 100,000 live births (LBs) in 2016, from 676 maternal deaths in 2011 [1, 2]. Infant mortality rate also halved from 97 deaths per 1000 LBs in 2000 to 48 deaths in 2016, and antenatal care by a skilled birth attendant increased from 27% in 2000 to 62% in 2016 [2]. This is partially due to sustained economic growth, a rapid increase of financial investments into the health system and moving away from an urban-centric model with greater emphasis on primary health care [3]. Nonetheless, more progress is needed if Ethiopia is to continue to improve health outcomes and sustain the progress made during the Millennium Development Goal era.

Ethiopia is focusing on providing universal access to quality health care without financial burden and ensuring an adaptive health system to meet the population’s changing health needs [4, 5]. Continued investment is needed to meet this goal and a substantial increase in domestic financing for health will allow for a more sustainable health system [6]. Understanding the costs of providing the current health service envelope is first needed to determine the resources that the government needs to mobilize for health, and to begin understanding the potential future costs of changes in quality of care, scope of service provision, and possible decline in external funding.

Few representative data on the costs or resources used to provide services at government-funded health care facilities exist for Ethiopia. One previous costing exercise in Ethiopia consisted of a very small sample of health facilities for pricing the potential social health insurance benefits package [7]. The results of that study do not reflect the significant changes to the health system since 2007. Other work includes normative costing exercises for the 5-year health sector plans and the essential health service package [8–10]. This type of costing is based on standards and norms to provide health care services but might not reflect real costs of service provision under field conditions.

Health service costing data have a variety of uses, such as health sector budgeting and planning, pricing of services, and reimbursement modalities for public sector services as part of insurance schemes. They can be valuable inputs in estimating service output efficiency, explaining causes of variations in cost/output ratios, identifying the right strategies to improve efficiency and quality, and support efforts to mobilize more domestic resources for health. We partnered with Ethiopia’s Ministry of Health (MoH) to conduct a primary health care service costing study in seven of the eleven regions in Ethiopia. The measurement of this type of data, and subsequent analyses that it can be used for, will contribute to Ethiopia’s health transformation agendas of equitable and quality of health care; improving data quality and use for effective decision-making; and woreda (district) transformation [4].

## Methods

### Study setting

Ethiopia’s government primary health care delivery organization is comprised of health posts, health centers, and primary hospitals. A health post, the lowest level of the primary health care system, mainly provides promotive and preventive health care services; serving 3000–5000 people in a woreda. A health center is a referral center for health posts, and provide promotive, preventive, curative and rehabilitative outpatient care including basic laboratory and pharmacy services; serving 15,000–25,000 people in a woreda. A primary hospital is the highest level of primary health care and provides inpatient and ambulatory services. This includes all of the same services offered at health centers, as well as additional emergency surgical services, including caesarian sections and blood transfusions, and serves as a referral center for health centers that reside within the primary hospital’s catchment area. Primary hospitals serve 60,000–100,000 people in a woreda. Investment in primary hospitals is still ongoing so not all health centers are linked with primary hospitals and some primary hospitals may serve several woredas [11, 12].

### Sample selection

The study was conducted in two phases in seven^1^ of the eleven regions of Ethiopia (Table 1). The first phase used a multi-stage cluster sampling to account for variation among regions and woredas with regard to major determinants that affect health service demand and utilization ensuring that units with particular characteristics (urban, rural, etc.) were included in the sample. Two regions, or city administrations, were selected within each stratified group. Three woredas were selected within each identified region, and in each selected woreda one primary hospital, three health centers (under the primary hospital catchment), and two health posts (under at least one of the health centers catchment) were selected for data collection. However, the inclusion of health posts for the analysis was not possible due to incomplete data records. Data collected during this phase was 2013/14 (the Ethiopian fiscal year (EFY) 2006). In the second phase of the study additional primary hospitals were purposively selected, using a proportional to population size sampling method among the four major regions – Tigray, Amhara, Oromia and Southern Nations, Nationalities, and Peoples’ Region (SNNPR) – where an adequate number of functioning primary hospitals were in existence for at least 1 year at the time of this supplemental data collection. Data collected during this phase was for 2014/15 (EFY 2007), with the exception of primary hospitals in Oromia which were not fully operational during this year. Data from primary hospitals in this region was collected from the last quarter of 2014/15 along with the first three quarters of 2015/16. No statistical difference was found among primary hospital facility characteristics and service statistics from 1 year to the next [11]. A total of 40 health centers and 24 primary hospitals had data of sufficient quality for analysis.

**Table 1:** Final sample for analysis by geographic location

	**Major Regions**				**Developing Regions**	**City Administration**		
**Health Facility Type**	Amhara	Oromia	SNNPR	Tigray	Benishangul-Gumuz	Addis Ababa	Dire Dawa^a^	**Total for Analysis**
Primary hospitals	8	8	5	3	0	0	0	24
Health Center	9	9	0	0	5	9	8	40
Total	17	17	5	3	5	9	8	64

Note: Data was collected from 94 PHC facilities (25 primary hospitals, 47 health centers, and 22 health posts). Poor data quality led to a reduction in the sample size from 94 to 64 PHC facilities during data analysis, where 1 primary hospital, 7 health centers, and 22 health posts had incomplete or poor data quality records beyond ‘fixing’ using extrapolation

^a^Dire Dawa sample selection process based on primary hospitals and health centers with data availability and accessibility (not based on woreda selection first since woredas do not exist in this city administration)

Secondary data including financial, administrative and health utilization (drug consumption, utilization rates, etc.) was extracted from the administrative records at the health facilities and other related health and financial institutions. Additionally, interviews with the health facility personnel were used to supplement data collection. A key informant interview guide was developed and used (Supplementary file 1). A paper-based survey instrument (Supplementary file 2) was used to extract the necessary data from the health facilities and other related institutions for this study.

### Costing methodology

The costing framework and analysis followed the major steps and recommendations outlined in guidelines by Hanson and Gilson, and Creese and Parker [13, 14] for conducting a cost accounting analysis in primary health care and hospitals (see Supplementary file 3). “Cost” is defined as the monetary value of non-capital, recurrent expenditures incurred, and resources used to produce a defined set of health service outputs or to operate specific health facilities. The recurrent costs include drugs and supplies, salaries, and other operational costs (e.g., electricity, running water, maintenance, etc.) incurred on a regular basis that were allocated as direct or indirect costs. Direct costs are costs directly attributable to a specific service output and consists of drugs and supplies and salaries. Indirect costs are other operational costs (e.g., electricity, running water, etc.) not attributed directly to a specific output. Both direct and indirect costs were included. Costs were estimated irrespective of where the money to cover such costs came from; such sources include revenue obtained through user fees, funds provided by federal block grants, and resources provided by development partners. Some primary care facilities rely on technical support from partners and nongovernmental organizations to administer certain exempted services. This study does not cost such technical assistance because this information is not collected and reported in the standard administrative records of the individual health facilities. The health facilities also do not record if the patients procure drugs and supplies from a private pharmacy outside the health facility, or the costs associated with patient referrals from one health facility to another. Thus, such costs were excluded from this analysis. Furthermore, capital costs were not included in this study due to study feasibility and other data limitations. Capital costs may not vary much by type of facility when amortized over long periods of time.

Direct and indirect costs were allocated across defined departments or cost centers for primary hospitals and health centers. The existing institutional arrangement for primary care services in Ethiopia was used to identify the four departments – Inpatient department (IPD), Outpatient department (OPD), Maternal and Child Health department (MCH), and Delivery department. Notably, the MCH department provides non-emergency maternal and child health services (such as immunizations, family planning, antenatal care, and post-natal care), excluding deliveries. On the other hand, the delivery department focuses on basic obstetric care (health centers) and comprehensive obstetric care (primary hospitals). Staff cost allocation was based on their formal work assignments because a detailed time-motion based allocation was not feasible. Key informant interviews at the facility were used to adjust any allocations of human resource (HR) costs where staff work in more than one department. A two-step approach was used to allocate the drugs and supplies costs by department. The first step consisted of focusing on *program* drugs and supplies that are used for specific treatments that would be offered under one of the four departments. The second step was to allocate drugs and supplies that could not be clearly allocated by department, consisting of *non-program* drugs and supplies for IPD, OPD, and Delivery department. The MCH department was not included because services offered at the MCH department are only considered as program-related services, such as family planning and vaccinations. For non-program drugs and supplies a ratio of 1:4 was used to allocate these costs across inpatient (IPD and Delivery) and outpatient departments [11]. This is a similar ratio found in paper by Özaltın and Cashin [15]. To allocate the non-program costs across the three departments, one-ninth of the costs went to OPD, four-ninths went to IPD, and the remaining four-ninths went to the Delivery department.

### Costing of exempted services

Exempted services are defined as the services that are offered for free to everyone among government-provided health facilities regardless of their income level [10]. Exempted services include care for tuberculosis, maternal and childcare (prenatal, delivery, postnatal, immunizations), family planning, antiretroviral treatment (ART) for HIV/AIDS, leprosy, and fistula and epidemics. A large portion of these services are financed by external funding, especially reproductive and maternal health [3]. Costing exempted services thus deserves special attention as the sustainability of these services is uncertain.

The costing framework for exempted services uses a similar approach as the primary hospitals and health centers departmental unit costs. Exempted services were classified under their respective departments (see Table 2). Some health centers do not offer all exempted services and thus omitted from such estimates. The HR and indirect costs were based on average per visit cost for all patients across the 4 defined departments. The unit costs for ANC and PNC were estimated using a cost-mix approach with normative and field-based costs because the drugs and supplies consumed for these two services could not be attributed directly to those particular services, unlike other exempted services such as malaria treatment or family planning.

**Table 2:** Costed exempted services by department

**Outpatient department (OPD)**	**Maternal and child health department (MCH)**	**Delivery department**
Tuberculosis	Expanded program for immunizations (all vaccines)	Delivery (natural and complicated)
Anti-retroviral treatment	Family planning	
Malaria	Antenatal care	
	Postnatal care	

### Unit cost output

Microsoft Excel 2010 and STATA 14 was used to analyse the data. Median estimates, instead of averages, are reported for unit and annual costs to minimize the effect of outliers.

The health facility unit cost is the ratio of the total recurrent costs relative to the total number of patient contacts for a given health facility. The department-wise unit cost is the ratio of total recurrent costs estimated for that department relative to the total patient contacts of that department.

Cost estimates are reported in both current Ethiopian birr (ETB) and purchasing power parity (PPP) with 2012 as the base year.

### Limitations

Readers should be cautious of the limitations of the study while interpreting the results. Unit cost estimates are affected by the quality of the available data. For example, number of patient contacts, the denominator for the unit cost estimates, was based on health management information systems data, which were at times found to be incomplete for the whole year and data imputations were used to fill data gaps. Also, problems were encountered during the data collection process, leading to a higher attrition of primary health care facilities in the study than expected and thus reducing our sample size from the originally planned 96 primary health care facilities to the 76 used in the costing analysis. See reference [11, 16] for more details of these issues. All operating costs for a primary health care facility could not be included in this study. It excludes capital costs,^2^ potential costs that are provided by technical assistance from development partners, and unforeseen additional costs to either a health provider or patient such as procuring drugs and supplies from a private pharmacy when stock-outs occur. Our analysis also does not reflect possible combined costs resulting from patient referrals from one health facility to another. Lastly, data issues to estimate exempted services limited disaggregating family planning services by specific types, determining the unit cost of treatment of leprosy, and led to a normative-based costing for ANC and PNC services rather than field representation. All of these limitations with this costing analysis either lead to overestimating (e.g., using normative costing instead of field-based costing for ANC and PNC) or underestimating (e.g., not including annual capital costs) costs for primary hospitals and health centers.

## Results

### Profile of study centers

The median catchment population for a health center and primary hospital in the study sample was 28,342 people and 352,805 people, respectively (Table 3). Most primary hospitals have a catchment population substantially higher than the standard (with a median almost 3.5 times more than the standard) since rollout of this relatively new health facility is still underway and typically serve multiple woredas.

**Table 3:** Health facility summary statistics (medians)

**Characteristic**	**Health centers (*N*** **= 40)**	**Primary Hospitals (*N*** **= 24)**
Catchment population	28,342	352,805
Annual per capita contact rate for catchment area population	0.805	0.660
Total staff count	47	154
Total clinical staff count^a^	20	63
Outpatient attendance per year	13,141	35,554
Total deliveries per year	239	744
Total IPD discharges per year	43	1104

^a^Clinical staff includes doctors, nurses, health officers, and midwives

Primary hospitals have about 3 times the clinical staff (doctors, nurses, health officers, and midwives) compared to health centers. The median per capita contact rate for health centers (0.805) is more than primary hospitals (0.660). This rate simply uses catchment population as the denominator and doesn’t account for possible spillover effects facilities may experience from other woredas.

### Annual costs

**Fig. 1:**
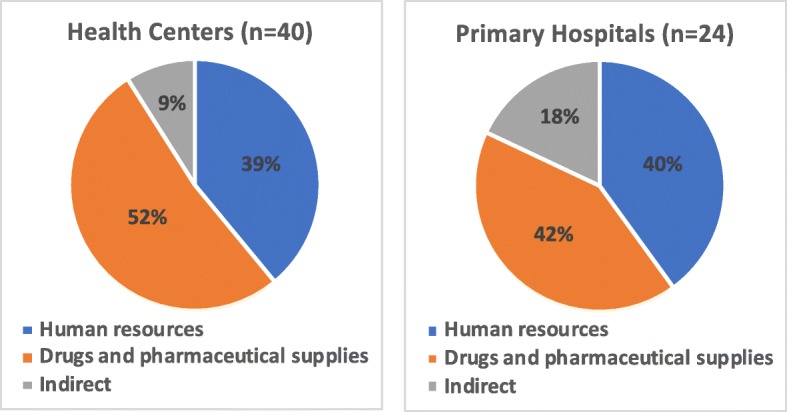
Percent distribution of annual median costs in ETB for health centers and primary hospitals. This figure presents the proportion of the cost components – human resources, drugs and pharmaceutical supplies, and indirect costs – from the total annual median costs for primary hospitals and health centers. The proportional break down of the median costs for health centers was: 52% for drugs and supplies, 39% for human resources, and the remaining 9% for indirect costs. The proportional break down of the median costs for primary hospitals was: 42% for drugs and supplies, 40% for human resources, and the remaining 18% for indirect costs. The sample size (n) for each health facility is presented – 40 health centers and 24 primary hospitals were used in the cost study

Referring to Table 4, the median annual recurrent costs for health centers and primary hospitals were ETB 3.9 million (0.46 million PPP $, 2012) and ETB 12 million (1.4 million PPP $, 2012), respectively. HR costs were comparable across the two health facilities, accounting for 39% for health centers and 40% for primary hospitals (Fig. 1). Drugs and supplies accounted for a substantial proportion of facility costs at 52% for health centers and 42% for primary hospitals.

**Table 4:** Annual costs of delivering healthcare services by health facility (median costs)

	**Health Centers (*n*** **= 40)**		**Primary Hopitals (*n*** **= 24)**	
**Annual Cost**	**ETB**	**PPP $, 2012**	**ETB**	**PPP $, 2012**
Drugs and pharmaceutical supplies	1,690,499	197,951	4,726,543	553,459
Human resources	1,241,826	145,413	4,483,344	524,982
Indirect expenditures	287,367	33,650	1,968,421	230,494
Total expenditure	3,924,119	459,499	12,012,302	1,405,152

*PPP* Purchasing power parity conversion factor = 8.54 Ethiopian birr to international dollar [17]

**Fig. 2:**
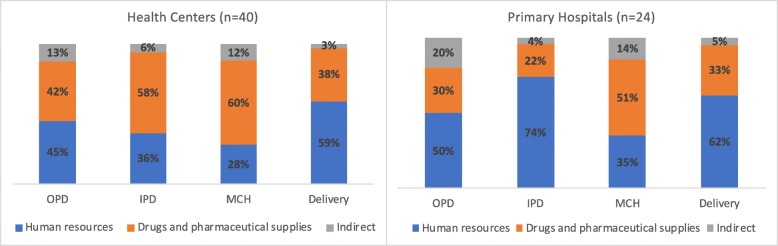
Percent share of total department expenditure in ETB for health centers and primary hospitals. This figure presents the proportion of the cost components for each health facility department – IPD, OPD, MCH, and Delivery. The main cost driver are drugs and supplies for health centers with the exception for the Delivery department and OPD. Indirect costs (e.g., electricity, running water, etc.) only account for a small portion of the total costs. We see a slightly different picture for primary hospitals where human resources are the main cost driver, accounting for at least 50% of total costs with the exception of the MCH department (35%)

The main cost driver were drugs and supplies for health centers with the exception for the Delivery department and OPD (Fig. 2). Both of these departments typically require more HR time for service provision, but the high HR costs might also be attributed to inefficiencies in human resource allocation based on patient load. Exploring this further, what “drives costs” is a combination of the costs and availability of drugs and supplies combined with the productivity of human resources. Unpacking this further requires additional analysis that is forthcoming. Indirect costs (e.g., electricity, running water, etc.) only accounted for a small portion of the total costs. We see a slightly different picture for primary hospitals where HR was the main cost driver, accounting for at least 50% of total costs with the exception of the MCH department (35%).

### Unit costs

Table 5 lists unit costs per service output per year for provision of various health services at the mentioned health facilities. The overall facility median unit cost for health centers was 146 ETB (17 PPP $, 2012), with the Delivery department having the highest median unit cost at 647 ETB (76 PPP $, 2012) and OPD with the lowest unit cost at 124 ETB (14 PPP $, 2012). The median health facility unit cost for primary hospitals was 339 ETB (40 PPP $, 2012), more than double compared to health centers. IPD had the highest median unit cost (1288 ETB (151 PPP $, 2012)), while OPD was the lowest (252 ETB (29 PPP $, 2012)) among primary hospitals.

**Table 5:** Unit costs of delivering health care services by health facility (median cost)

	**Health Center Unit Costs (*N*** **= 40)**		**Primary Hospitals Unit Costs (*N*** **= 24)**	
**Facility/Department Type**	**ETB**	**PPP $, 2012**	**ETB**	**PPP $, 2012**
Health facility	146	17	339	40
OPD	124	14	252	29
IPD^a^	340	40	1288	151
MCH	145	17	310	36
Delivery	647	76	945	111

^a^Sample for IPD unit costs for the health centers was reduced from 40 to 30 because only 30 health centers had IPDs

### Regional distribution of unit costs

**Fig. 3:**
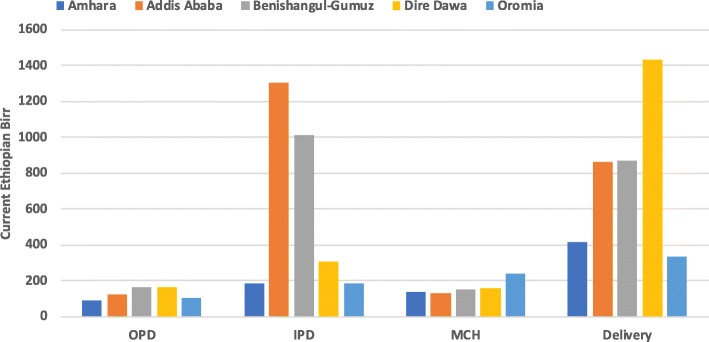
Regional distribution of median unit costs in ETB by department for health centers. This figure shows the regional distribution of the median unit costs (in Ethiopian birr) across the 4 departments for health centers. OPD and MCH had substantially lower median unit costs compared to IPD and Delivery department across all regions. The lower median unit costs in certain regions could be due to cost-efficient use of resources, high utilization rates, or even inefficiencies not captured by this analysis (such as low expenditures due to high stock-out rates). The highest median unit costs were for inpatient services provided in Addis Ababa and delivery services provided in Dire Dawa. Both Addis Ababa and Dire Dawa are urban areas

**Fig. 4:**
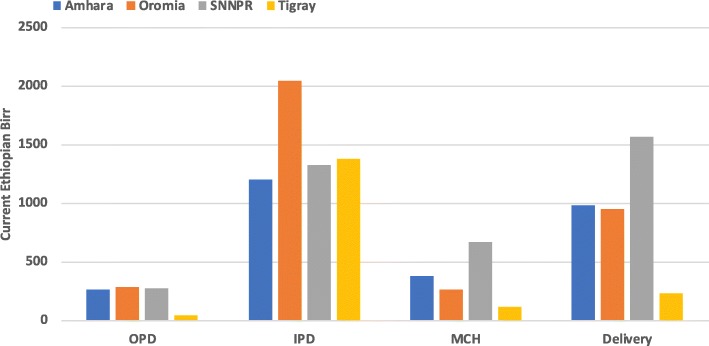
Regional distribution of median unit costs in ETB by department for primary hospitals. This figure shows the regional distribution of the median unit costs (in Ethiopian birr) across the 4 departments for primary hospitals. Overall, the lowest median unit costs for almost all regions were for outpatient and maternal and child health services. The lower median unit costs in certain regions could be due to cost-efficient use of resources, high utilization rates, or even inefficiencies not captured by this analysis (such as low expenditures due to high stock-out rates). The highest median unit costs were inpatient services provided in Oromia primary hospitals and delivery services provided in SNNPR primary hospitals

The distribution of median unit costs varied significantly by region across the studied health centers, ranging from 116 to 171 ETB (14–20 PPP $, 2012), and primary hospitals, ranging from 71 to 457 ETB (8–53 PPP$, 2012) (Table 6). Across all the regions, OPD and MCH had substantially lower median unit costs compared to both IPD and Delivery department (Figs 3 and 4).

**Table 6:** Regional distribution of median unit costs by department for each health facility type

	**Health Centers**		**Primary Hospitals**	
**Regions**	**ETB**	**PPP$, 2012**	**ETB**	**PPP$, 2012**
Amhara	116	14	336	39
Addis Ababa	145	17	na	na
Benishangul-Gumuz	161	19	na	na
Dire Dawa	158	19	na	na
Oromia	171	20	353	41
SNNPR	na	na	457	53
Tigray	na	na	71	8

*na* not applicable; data was not collected in the region for that health facility

### Unit cost of exempted services

Table 7 shows the estimated median unit costs for exempted services (health services that are free to all regardless of welfare) in both ETB and PPP $ (2012) for the studied primary hospitals and health centers.

**Table 7:**
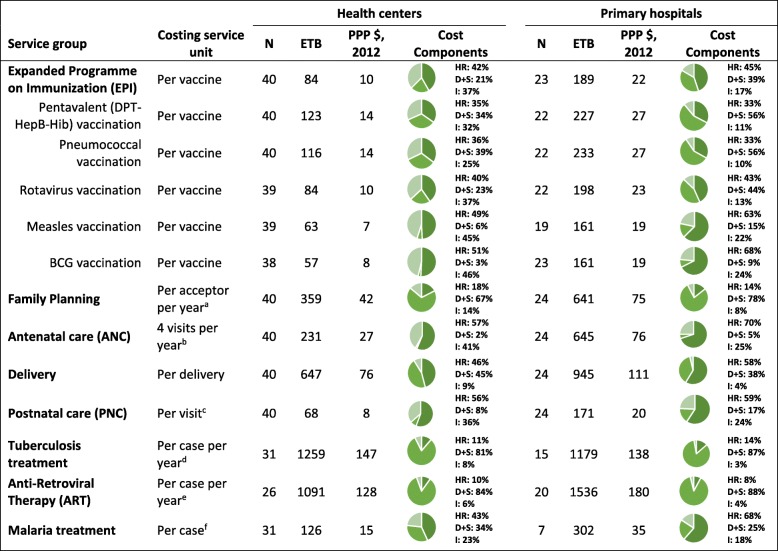
Median unit cost and cost components of exempted services by health facility

Source: Berman et al. 2016. **HR: Human resources D + S: Drugs and pharmaceutical supplies I: Indirect**

^a^An acceptor consists of new and repeat patients, from the previous fiscal year, of reproductive age (15–49 years) that receives a modern contraceptive service in the calendar year

^b^At least four ANC visits during the EFY

^c^A PNC visit within 24 h of a newborn’s birth during the EFY

^d^Tuberculosis case (all forms) that was registered at each facility for the EFY.

^e^A person living with HIV/AIDS (adult or child) that received ART for the EFY, with assumption that an individual did not stop treatment or miss regimens throughout the year

^f^New malaria case (complicated or severe) registered at the health center during the EFY

#### Unit cost for EPI

The median recurrent unit cost for the EPI vaccines administered at health centers and primary hospitals was 84 ETB (10 PPP $, 2012) and 189 ETB (22 PPP $, 2012) per vaccination, respectively. Among primary hospitals, the largest share of this cost comes from HR (42%), followed by indirect costs (37%), and drugs and supplies (21%). A majority of the EPI costs for health centers is comprised of HR (45%), followed by drugs and supplies (39%), and indirect costs (17%). Looking more closely at the individual vaccines under this program, the median unit costs vary. For the health centers, the measles vaccine had the lowest median cost to administer (63 ETB (7 PPP $, 2012) per vaccination), while the pentavalent vaccine had the highest median cost to administer (123 ETB (14 PPP $, 2012) per vaccination). Among these primary hospitals, the Pneumococcal vaccine had the highest median cost to administer (233 ETB (27 PPP $, 2012) per vaccination), while the BCG and measles vaccine had the lowest median cost to administer (161 ETB (19 PPP $, 2012) per vaccination each).

#### Unit cost for family planning services

An “acceptor” for family planning is a patient of reproductive age (15–49 years) receiving a modern contraceptive method. Contraceptive services include provision of contraceptive supplies as well as routine check-ups for ongoing use of a long-term method such as Norplant, intrauterine device (IUD), etc. The median recurrent unit cost for a family planning service was moderately high for both health centers and primary hospitals, at 359 ETB (42 PPP $, 2012) and 641 ETB 75 PPP $, 2012) per acceptor per year, respectively. Drugs and supplies, which constitute of birth control commodities (pills, injectables, and IUD), condoms, and emergency contraception, were the major cost drivers for family planning services at both primary hospitals (67%) and health centers (78%).

#### Unit cost for ANC, delivery, and PNC

ANC unit costs were based on at least 4 ANC visits per year, while PNC unit costs were based on a typical post-partum visit within the first 24 h of a newborn’s birth during the study period. ANC services typically included regular check-ups, tetanus toxoid shots, and/or syphilis detection and treatment. The median unit cost for 4 ANC visits among health centers was 231 ETB (27 PPP $, 2012) and 645 ETB (76 PPP $, 2012) for primary hospitals. PNC services consisted of treatments for newborn sepsis, treatment of postpartum hemorrhage, and prescribing Chlorhexidine for treatment of the umbilical cord among newborns, among others. The median unit cost of an average PNC service among health centers and primary hospitals was 68 ETB (8 PPP $, 2012) and 171 ETB (20 PPP $, 2012), respectively. The delivery median unit costs were based on a normal vaginal delivery conducted at the health facility and were estimated at 647 ETB (76 PPP $, 2012) for health centers and 945 ETB (111 PPP $, 2012) for primary hospitals. The major cost driver for a delivery was HR costs at both health centers (59%) and primary hospitals (62%).

#### Unit cost for tuberculosis, ART and Malaria treatment

The median recurrent unit cost per tuberculosis case per year was 1259 ETB (147 PPP $, 2012) for a health center and 1179 ETB (138 PPP $, 2012) for primary hospitals. Drugs and supplies were the main cost drivers for this treatment for both primary hospitals (81%) and health centers (87%). ART median unit cost estimates were based on the number of people living with HIV/AIDS currently receiving ART for EFY 2006, and was estimated at 1091ETB (128 PPP $, 2012) and 1536 ETB (180 PPP $, 2012) per case per year for health centers and primary hospitals, respectively. A majority of ART costs were for drugs and supplies, consisting of 84% of the median unit costs for primary hospitals and 88% for health centers. Malaria treatment included both complicated or severe cases (infections complicated by organ failure or abnormalities in patient’s blood or metabolism) and uncomplicated cases (malaria attack that lasts 6–10 h) [9, 18]. Uncomplicated cases would have a lower unit cost estimate compared to complicated ones, however the distinction between these two types of malaria cases was not possible for this analysis. The median unit cost for malaria treatment was 126 ETB (15 PPP $, 2012) per case per year for health centers and 302 ETB (35 PPP $, 2012) for the primary hospitals. HR were the main cost driver for malaria treatment at 43 and 68% in primary hospitals and health centers, respectively.

## Discussion

This paper provides an estimate of the total annual and unit costs for Ethiopian government-funded primary health care facilities, departments, and exempted services. Spending on primary health care services are relatively high when considering them within the overall low total government per capita expenditure of ETB 169 (20 PPP $, 2012) (2013/14) excluding external sources [19]. These are also high compared to other Sub-Saharan African countries [20, 21], and given that Ethiopia is still a relatively low-income country [22]. High unit costs might be due to a high number of staff relative to demand or inefficiencies in resource use driving up the costs. The former could be reasonable in a rapidly growing system, which anticipates significant future increases in demand and is taking proactive measures to be prepared for that to happen [23]. Also, possible stock-outs may lead primary care facilities to procure drugs and supplies from costlier private pharmacies.

Moreover, an estimated 73% of government primary health care spending is from external funding [6]. Under the current circumstances of uncertainty in the flow of external funding, it will be difficult for Ethiopia to maintain business as usual in domestic health spending and sustain these high unit costs of government-funded service provision [6].

Drugs and pharmaceutical supplies are the highest cost component for health centers (52%) and primary hospitals (42%), which is substantially higher compared to other low-and-middle income countries’ (LMICs’) primary care facilities where the share of personnel costs was over 50% [24–26]. A systematic review found that the availability of quality drugs is an indicator of the difference in quality of private versus public ambulatory health care in LMICs [27]. Thus, spending more on drugs and supplies might be encouraging as it may reflect adequate spending on supplies needed to deliver quality health services at publicly funded facilities. However, it might also indicate inefficiencies with possible losses due to improper storage and expiration or even low quality of care with limited higher-paid health staff (e.g., doctors).

Funding for exempted services is provided by either external resources or from the government, as they are to be *exempt* from individuals paying user fees for these services. External support for these services is mostly in-kind, providing a majority of the drugs and supplies. Drugs and pharmaceutical supplies are the main cost driver, meaning the domestic health budget would have to increase substantially to at least maintain the current exempted service package, should external support decline due to continued rapid economic development, changing global financial landscape, and additional competing priorities with the Sustainable Development Goals (SDGs). One of the most expensive exempted services was ART and almost 90% of the cost is from drugs and supplies. With the relatively recent cuts in the United States President’s Emergency Plan for AIDS Relief (PEPFAR) funding for Ethiopia [6], the government has to cover these high costs to ensure that such services continue for free.

Unit cost results show some patterns of variation between facility types and location. For example, health centers in rural areas have lower department-level unit costs, as compared to the urban, except for the MCH departments. A variety of factors, including quality of care, cost implications due to stock-outs, or additional costs incurred because of remoteness (e.g., additional transportation costs to delivery drugs and supplies to more remote health facility) may contribute to such differences. The lower median unit costs in certain regions could be due to cost-efficient use of resources, high utilization rates, or even inefficiencies not captured by this analysis (such as low expenditures due to high stock-out rates).

The Ethiopian health system is undergoing a transition in order to be more responsive to the changing population health needs. This includes expanding primary hospital coverage, increasing funding of exempted services from domestic resources, and revising the essential health services package list. The cost estimates in this study provide critical data for policy makers and health sector managers to support their financial planning for such changes.

Addressing major data limitations of this study would further refine future unit cost estimates for primary care service provision. Data quality and availability were the largest hurdles faced during data collection and analysis. Although the MoH has in place a good system of reporting and recording of input- and output-related data, we often found these data to be incomplete or not available at facility level. For instance, the estimation of program drugs consumption was done using the monthly Report and Requisition Forms (RRFs). Many of the health facilities did not have a completed set of RRFs pertaining to the study period. In such cases, extrapolations were carried out using the available RRFs. This can potentially affect the validity of drug consumption estimates. Similarly, service volume statistics were obtained from the health management information system (HMIS). Data were extrapolated to estimate the service volume for those health facilities where completed HMIS reports were not available for the entire duration of the study period. To strengthen health system performance for quality and efficiency, more effort will be needed to assure that these essential data are maintained and used. This is one of the key goals of Ethiopia’s Health Sector Transformation Plan (HSTP, 2015/16–2019/20) in its transformational agenda for an “information revolution” [4]. The relatively recent roll-out of the District Health Information Software 2 across Ethiopia is seen as a pathway to improve data quality and increase use. Additionally, better record keeping and regular audits of primary health care facilities could help in minimizing potential estimation biases resulting from extrapolated data.

## Conclusion

This study reports empirical cost data for Ethiopia’s government-provided primary health care system. High unit costs of service provision could be indicative of underutilization of the primary health care system, coupled with inefficiencies associated with organization and delivery of health services. The data from this study could be further used to assess efficiency and productivity among the primary care facilities, facilitate premium setting for health insurance, and improve budgeting and allocating health resources for a more sustainable and effective primary health care system. Based on this study’s findings more work is needed to understand contributing factors of potential resource input inefficiencies, causes of regional variations in unit costs, and explanations for the low level of personnel costs relative to drugs and supplies.

The data intensive nature of costing exercises also points to the importance of maintaining records and data systems at the facility level to enable accuracy and replicability of future studies, for which our study could serve as a baseline.

## Supplementary information

**Additional file 1: Supplementary file 1.** Key informant interview guide. This is the key informant guide that was used to conduct interviews with health facility heads or top management at health facilities in the study sample. This interview was to capture additional information on resource allocation and use challenges, identified solutions, and best practices to supplement data collection at the health facilities.

**Additional file 2: Supplementary file 2.** Cost study instruments. This paper-based survey instrument was used to extract the necessary data from the health facilities and other related institutions to capture relevant data for this study such as service statistics, drugs and supplies consumed, human resource data including salaries, etc.

**Additional file 3: Supplementary file 3.** Cost accounting steps. This document lays out the specific cost accounting steps used in this costing study. This consists of description of each of the six steps taken: 1) Define the final product; 2) Define the cost centers; 3) Identify and allocate direct costs; 4) Identify and allocate indirect costs; 5) Allocate all costs to cost center; and 6) Compute total and average costs for each final cost center.

## Data Availability

The instruments used to collect the data at the primary health care facilities to conduct the costing study presented in this article are included in this article (Supplementary files 1 and 2). The data used and analysed for this study are available from the corresponding author on reasonable request.

## Abbreviations

ANC: Antenatal care
ART: Antiretroviral treatment
BCG: Bacille Calmette-Guerin
EPI: Expanded program for immunizations
ETB: Ethiopian birr
EFY: Ethiopian fiscal year
MCH: Maternal and child health
HR: Human resources
NGO: Non-governmental organization
LB: Live births
SNNPR: Southern Nations, Nationalities, and Peoples' Region
IPD: Inpatient department
MoH: Ministry of Health
PEPFAR: The United States President’s Plan for AIDS Relief
PNC: Postnatal care
OPD: Outpatient department
PPP: Purchasing power parity
HSTP: Health Sector Transformation Plan
LMIC: Low-and-middle income countries
SDGs: Sustainable development goals
RRF: Report and requisition form

## Footnotes

1 Somali region was initially included to add to the representation of the developing regions; however, data was very limited and aggregated at the woreda level rather than facility level. Therefore, facilities from this region could not be included in the study.

2 Capital costs may not vary much by type of facility when amortized over long periods of time. These costs could be added later from other data sources on inputs and their costs.
