# Supplementary material for: Recurrent costs in primary health care in Ethiopia: facility and disease specific unit costs and their components in government primary hospitals and health centers
Source: BMC Health Serv Res. 2020 May 7;20:389. doi: 10.1186/s12913-020-05218-1 (PMC7204209; doi:10.1186/s12913-020-05218-1)
Supplement: Supplementary file 1 — Additional file 1: Supplementary file 1. Key informant interview guide. This is the key informant guide that was used to conduct interviews with health facility heads or top management at health facilities in the study sample. This interview was to capture additional information on resource allocation and use challenges, identified solutions, and best practices to supplement data collection at the health facilities. [file 12913_2020_5218_MOESM1_ESM.docx]

**Supplementary file 1: Key Informant Interview Guide**

## Instruction

The FMOH in collaboration with Harvard School of Public Health (HSPH) is implementing Resource Tracking and Management (RTM) project. One component of the Resource Tracking and Management (RTM) project in Ethiopia focuses on improving understanding of variability of resource utilization and productivity in primary care services and helping to identify measures that could be employed to improve it as a means of improving health services performance. This questionnaire is prepared to assess and capture relevant data with this regard.

## Respondent Information

- 1. Region _____________________________
  2. Zone _____________________________
  3. Woreda _____________________________
  4. Respondent is from:
     1. Zonal/Woreda Health Office
     2. Health Facility

If Health Facility

- 1. Facility Type _____________________________
  2. Name of Facility _____________________________

## Interview Information

- 1. Date of Interview _________________
  2. Name of interviewer _____________________________
  3. Name and contact address of the main respondents

| No | Name | Responsibility | Contact details |
| --- | --- | --- | --- |
|  |  |  |  |
|  |  |  |  |
|  |  |  |  |
|  |  |  |  |

# Checklist for KII

**Note: You should ask the facility head or top management for this part**

1. Would you share us your best practices and challenges in relation to resource allocations at the facilities level? (probe with regard to)
   1. Human resources
   2. Drugs and supplies
   3. Equipment
   4. Finance
   5. Other
2. Do you feel that you have adequately (optimally) utilized available resources at your Facility (probe with regard to)
   1. Human resource
   2. Equipment
   3. Beds
   4. Medical Supplies
   5. Budget
   6. Other support from partners
3. Are there major actions carried out by the government or other players to address each of the critical resource allocation challenges identified?
4. What solutions do you suggest to overcome non-optimal resource allocations?
5. What type of support should be sought after from country counterparts for the solution?
