# Supplementary material for: Recurrent costs in primary health care in Ethiopia: facility and disease specific unit costs and their components in government primary hospitals and health centers
Source: BMC Health Serv Res. 2020 May 7;20:389. doi: 10.1186/s12913-020-05218-1 (PMC7204209; doi:10.1186/s12913-020-05218-1)
Supplement: Supplementary file 2 — Additional file 2: Supplementary file 2. Cost study instruments. This paper-based survey instrument was used to extract the necessary data from the health facilities and other related institutions to capture relevant data for this study such as service statistics, drugs and supplies consumed, human resource data including salaries, etc. [file 12913_2020_5218_MOESM2_ESM.docx]

**Supplementary file 2: Cost Study Instruments**

**Cost Analysis of Service Delivery at PHC Level in Ethiopia**

**March 2015**

**Study instruments**

**HEALTH CENTERS AND HOSPITALS**

| 1. **COVER SHEET** | | |
| --- | --- | --- |
|  | **FACILITY IDENTIFICATION** |  |
| 1.1 | Name of health facility | _________________________________________ |
| 1.2 | Type of health facility  ***01 = Hospital 02 = Health center*** | \|  \|  \| \| --- \| --- \| |
| 1.3 | Region |  |
| 1.4 | Zone |  |
| 1.5 | Woreda |  |
| 1.6 | Geographical classification  ***01 = Rural 02 = Urban*** | \|  \|  \| \| --- \| --- \| |
|  |  |  |
|  | **KEY CONTACT INFORMATION (Facility In Charge)** |  |
| 1.7 | Name of the person |  |
| 1.8 | Position / Job title in health facility |  |
| 1.9 | Telephone number of the person |  |
| 1.10 | Email of the person (if any) |  |
|  |  |  |
|  | **FACILITY BACKGROUND INFORMATION** |  |
| 1.11 | Total catchment population |  |
| 1.12 | Total number of attached health posts  (only for health centers) | \|  \|  \| \| --- \| --- \| |
| 1.13 | Year of establishment of health facility (E.C.) |  |
|  |  |  |

**COMMENTS BOX: Record general notes about the interview and any special information that will be helpful for supervisors, office editors, and data analysts**

|  |
| --- |

1. **PROCESS MONITORING**

|  |  |  |  |  |  |  |  |  |  | | |  | |  | |  |  |  |
| --- | --- | --- | --- | --- | --- | --- | --- | --- | --- | --- | --- | --- | --- | --- | --- | --- | --- | --- |
|  | **2.1 FIELD DATA COLLECTION PHASE (To be filled by the field supervisor)** | | | | | | | | | | | | | | |  |  |  |
|  |  |  |  |  |  |  |  |  |  | | |  | |  | |  |  |  |
|  | 2.1.1 | Date of interview |  |  |  |  |  |  |  |  | |  | | |  | |  |  |
|  |  |  | D | D |  | M | M |  | Y | | | Y | | Y | | Y |  |  |
|  |  |  |  |  |  |  |  |  |  | | |  | |  | |  |  |  |
|  | 2.1.2 | Interviewer’s ID number |  |  |  |  |  |  |  | | |  | |  | |  |  |  |
|  |  |  |  |  |  |  |  |  |  | | |  | |  | |  |  |  |
|  |  |  |  |  |  |  |  |  |  | | |  | |  | |  |  |  |
|  | 2.1.3 | Supervisor’s name and ID number | ________________________ | | | | |  |  | |  | |  | | |  |  |  |
|  |  |  |  | | | | | | | | | | | | | |  |  |
|  |  |  |  |  |  |  |  |  |  | | |  | |  | |  |  |  |
|  | **2.2 OFFICE EDITING PHASE (To be filled by the office editing supervisor)** | | | | | | | | | | | | | | |  |  |  |
|  |  |  |  |  |  |  |  |  |  | | |  | |  | |  |  |  |
|  | 2.2.1 | Date of office editing |  |  |  |  |  |  |  |  | |  | | |  | |  |  |
|  |  |  | D | D |  | M | M |  | Y | | | Y | | Y | | Y |  |  |
|  |  |  |  |  |  |  |  |  |  | | |  | |  | |  |  |  |
|  | 2.2.2 | Office editor’s name and ID number | ________________________ | | | | |  |  | |  | |  | | |  |  |  |
|  |  |  |  | | | | | | | | | | | | | |  |  |
|  |  |  |  |  |  |  |  |  |  | | |  | |  | |  |  |  |
|  | **2.3 DATA ENTRY PHASE (To be filled by the data entry supervisor)** | | | | | | | | | | |  | |  | |  |  |  |
|  |  |  |  |  |  |  |  |  |  | | |  | |  | |  |  |  |
|  | 2.3.1 | Date of interview |  |  |  |  |  |  |  |  | |  | | |  | |  |  |
|  |  |  | D | D |  | M | M |  | Y | | | Y | | Y | | Y |  |  |
|  |  |  |  |  |  |  |  |  |  | | |  | |  | |  |  |  |
|  | 2.3.2 | Data entry clerk’s name and ID number | ________________________ | | | | |  |  | |  | |  | | |  |  |  |
|  |  |  |  | | | | | | | | | | | | | |  |  |
|  |  |  |  |  |  |  |  |  |  | | |  | |  | |  |  |  |

1. **PERSONNEL COSTS**

Instructions:

1. The source of information for this data is the monthly payroll sheet available in the Finance department of the health facility (for example, Form 7). If not, get this information from the Woreda finance.
2. Include both permanent and non-permanent (contracted) staff.
3. Collect this information for the month of Sene 2006. In case, the information is not available for Sene 2006, request the same for the last available month for the fiscal year 2006. Make a note of this change in the comments box of this form.
4. Monthly salary refers to the gross salary (before deductions of taxes and pension)
5. If the payroll sheet does not clearly identify the positions (3.4) of staff, ask for this information from the administration / human resource (HR) department.
6. Identify all staff in the payroll of the health facility, but are assigned to the associated health posts. Write “X” for all staff identified as health posts staff in column 3.5. This information can be obtained from the HR department.
7. Allowances (3.7) can be of different types. From the sheet where staff allowances are recorded, please add them together and then write the total figure for each staff (use a calculator to add them together, in case the total figure is not already written).
8. Duty payments (3.10 – 3.15) is recorded in a separate form in the finance department of health facility or Woreda finance. Record the figures for the months mentioned in 3.10 – 3.15. In case these figures for the mentioned months are not available, replace it with another month in the same quarter. Make a note of this change in the comments box of this form.
9. In the case of duty payables that are approved regarding rates and duration but amount due are not calculated or paid ask the finance officer to assist in calculating the amount due and record; write the situation in the comment box (probable cases in Amhara Region for the months of Hamle and Nehase 2005; could also encounter in other areas as well).
10. If the number of staff in payroll is more than 30, use extra sheets. Please record the total number of sheets used in the place provided.
11. Ask the HR if there are people on the payroll who have been absent due to studying or other reasons for the period (2006 FY) and write in the comment box.
12. No cells should be left blank. If the figure for a particular cell is not available, write “**missing**” clearly. If it’s a zero, write “0”.

| 1. **PERSONNEL COSTS (Salary, wages, benefits) 3.1 Sheet \|0\|1\| of \|__\|__\|** | | | | | | | | | | | | | |
| --- | --- | --- | --- | --- | --- | --- | --- | --- | --- | --- | --- | --- | --- |
|  | **STAFF IDENTIFICATION** | | | **COMPENSATION DETAILS** | | | | | | | | | |
| **3.2**  **S. No.** | **3.3 Name** | **3.4 Position or Job title** | **3.5 Health post staff?** | **REGULAR PAYMENTS** | | | | **DUTY PAYMENTS** | | | | | |
|  |  |  |  | **F.Y. 2006** | | | | **F.Y. 2006** | | | | **F.Y. 2007** | |
|  |  |  |  | **3.6 Monthly salary** | **3.7 Allowances** | **3.8**  **Total Payable** | **3.9 Other payment (if any)** | **3.10**  **Nehase 2005** | **3.11**  **Hidar 2006** | **3.12**  **Tirr**  **2006** | **3.13**  **Megabit 2006** | **3.14**  **Nehase 2006** | **3.15**  **Hidar 2007** |
| 1 |  |  | \|  \| \| --- \| |  |  |  |  |  |  |  |  |  |  |
| 2 |  |  | \|  \| \| --- \| |  |  |  |  |  |  |  |  |  |  |
| 3 |  |  | \|  \| \| --- \| |  |  |  |  |  |  |  |  |  |  |
| 4 |  |  | \|  \| \| --- \| |  |  |  |  |  |  |  |  |  |  |
| 5 |  |  | \|  \| \| --- \| |  |  |  |  |  |  |  |  |  |  |
| 6 |  |  | \|  \| \| --- \| |  |  |  |  |  |  |  |  |  |  |
| 7 |  |  | \|  \| \| --- \| |  |  |  |  |  |  |  |  |  |  |
| 8 |  |  | \|  \| \| --- \| |  |  |  |  |  |  |  |  |  |  |
| 9 |  |  | \|  \| \| --- \| |  |  |  |  |  |  |  |  |  |  |
| 10 |  |  | \|  \| \| --- \| |  |  |  |  |  |  |  |  |  |  |
| 11 |  |  | \|  \| \| --- \| |  |  |  |  |  |  |  |  |  |  |
| 12 |  |  | \|  \| \| --- \| |  |  |  |  |  |  |  |  |  |  |
| 13 |  |  | \|  \| \| --- \| |  |  |  |  |  |  |  |  |  |  |
| 14 |  |  | \|  \| \| --- \| |  |  |  |  |  |  |  |  |  |  |
| 15 |  |  | \|  \| \| --- \| |  |  |  |  |  |  |  |  |  |  |
| 16 |  |  | \|  \| \| --- \| |  |  |  |  |  |  |  |  |  |  |
| 17 |  |  | \|  \| \| --- \| |  |  |  |  |  |  |  |  |  |  |
| 18 |  |  | \|  \| \| --- \| |  |  |  |  |  |  |  |  |  |  |
| 19 |  |  | \|  \| \| --- \| |  |  |  |  |  |  |  |  |  |  |
| 20 |  |  | \|  \| \| --- \| |  |  |  |  |  |  |  |  |  |  |
| 21 |  |  | \|  \| \| --- \| |  |  |  |  |  |  |  |  |  |  |
| 22 |  |  | \|  \| \| --- \| |  |  |  |  |  |  |  |  |  |  |
| 23 |  |  | \|  \| \| --- \| |  |  |  |  |  |  |  |  |  |  |
| 24 |  |  | \|  \| \| --- \| |  |  |  |  |  |  |  |  |  |  |
| 25 |  |  | \|  \| \| --- \| |  |  |  |  |  |  |  |  |  |  |
| 26 |  |  | \|  \| \| --- \| |  |  |  |  |  |  |  |  |  |  |
| 27 |  |  | \|  \| \| --- \| |  |  |  |  |  |  |  |  |  |  |
| 28 |  |  | \|  \| \| --- \| |  |  |  |  |  |  |  |  |  |  |
| 29 |  |  | \|  \| \| --- \| |  |  |  |  |  |  |  |  |  |  |
| 30 |  |  | \|  \| \| --- \| |  |  |  |  |  |  |  |  |  |  |
| 1. **PERSONNEL COSTS (Salary, wages, benefits) 3.1 Sheet \|__\|__\| of \|__\|__\|** | | | | | | | | | | | | | |
|  | **STAFF IDENTIFICATION** | | | **COMPENSATION DETAILS** | | | | | | | | | |
| **3.2**  **S. No.** | **3.3 Name** | **3.4 Position or Job title** | **3.5 Health post staff?** | **REGULAR PAYMENTS** | | | | **DUTY PAYMENTS** | | | | | |
|  |  |  |  | **F.Y. 2006** | | | | **F.Y. 2006** | | | | **F.Y. 2007** | |
|  |  |  |  | **3.6 Monthly salary** | **3.7 Allowances** | **3.8**  **Total Payable** | **3.9 Other payment (if any)** | **3.10**  **Nehase 2005** | **3.11**  **Hidar 2006** | **3.12**  **Tirr**  **2006** | **3.13**  **Megabit 2006** | **3.14**  **Nehase 2006** | **3.15**  **Hidar 2007** |
|  |  |  | \|  \| \| --- \| |  |  |  |  |  |  |  |  |  |  |
|  |  |  | \|  \| \| --- \| |  |  |  |  |  |  |  |  |  |  |
|  |  |  | \|  \| \| --- \| |  |  |  |  |  |  |  |  |  |  |
|  |  |  | \|  \| \| --- \| |  |  |  |  |  |  |  |  |  |  |
|  |  |  | \|  \| \| --- \| |  |  |  |  |  |  |  |  |  |  |
|  |  |  | \|  \| \| --- \| |  |  |  |  |  |  |  |  |  |  |
|  |  |  | \|  \| \| --- \| |  |  |  |  |  |  |  |  |  |  |
|  |  |  | \|  \| \| --- \| |  |  |  |  |  |  |  |  |  |  |
|  |  |  | \|  \| \| --- \| |  |  |  |  |  |  |  |  |  |  |
|  |  |  | \|  \| \| --- \| |  |  |  |  |  |  |  |  |  |  |
|  |  |  | \|  \| \| --- \| |  |  |  |  |  |  |  |  |  |  |
|  |  |  | \|  \| \| --- \| |  |  |  |  |  |  |  |  |  |  |
|  |  |  | \|  \| \| --- \| |  |  |  |  |  |  |  |  |  |  |
|  |  |  | \|  \| \| --- \| |  |  |  |  |  |  |  |  |  |  |
|  |  |  | \|  \| \| --- \| |  |  |  |  |  |  |  |  |  |  |
|  |  |  | \|  \| \| --- \| |  |  |  |  |  |  |  |  |  |  |
|  |  |  | \|  \| \| --- \| |  |  |  |  |  |  |  |  |  |  |
|  |  |  | \|  \| \| --- \| |  |  |  |  |  |  |  |  |  |  |
|  |  |  | \|  \| \| --- \| |  |  |  |  |  |  |  |  |  |  |
|  |  |  | \|  \| \| --- \| |  |  |  |  |  |  |  |  |  |  |
|  |  |  | \|  \| \| --- \| |  |  |  |  |  |  |  |  |  |  |
|  |  |  | \|  \| \| --- \| |  |  |  |  |  |  |  |  |  |  |
|  |  |  | \|  \| \| --- \| |  |  |  |  |  |  |  |  |  |  |
|  |  |  | \|  \| \| --- \| |  |  |  |  |  |  |  |  |  |  |
|  |  |  | \|  \| \| --- \| |  |  |  |  |  |  |  |  |  |  |
|  |  |  | \|  \| \| --- \| |  |  |  |  |  |  |  |  |  |  |
|  |  |  | \|  \| \| --- \| |  |  |  |  |  |  |  |  |  |  |
|  |  |  | \|  \| \| --- \| |  |  |  |  |  |  |  |  |  |  |
|  |  |  | \|  \| \| --- \| |  |  |  |  |  |  |  |  |  |  |
|  |  |  | \|  \| \| --- \| |  |  |  |  |  |  |  |  |  |  |

1. **MONTHLY AGGREGATES OF PERSONNEL COSTS IN HEALTH FACILITY FOR FISCAL YEAR 2005-06**

**Instructions:**

1. Data for 4.2, 4.3 and 4.4 (shaded in dark) comes from the payroll sheets of each month (for F.Y. 2005-06), available in the Finance department, or Woreda finance office. The last row in the payroll sheet contains these total figures. In case the summation is not available, add the relevant figures for all staff for each month.
2. The Finance department records the duty payments (4.5) for each month separately from the payroll.
3. Enquire about any other payments (4.6) made to the staff employed by the health facility and note down in the relevant months

No cells should be left blank. If the figure for a particular cell is not available, write “**missing**” clearly. If it’s a zero, write “0”.

|  |  |  |  | **RECORD THE FOLLOWING FIGURES AS THE TOTAL FOR THE MONTH (IN BIRR)** | | | | | | | | | | | | | | | | | | | | | | | | | | | | | | | | | | |
| --- | --- | --- | --- | --- | --- | --- | --- | --- | --- | --- | --- | --- | --- | --- | --- | --- | --- | --- | --- | --- | --- | --- | --- | --- | --- | --- | --- | --- | --- | --- | --- | --- | --- | --- | --- | --- | --- | --- |
| **Month** | **4.1 Total number of staff** | | | **4.2 Total of monthly salary** | | | | | | | **4.3 Total of Allowances** | | | | | | | **4.4 Total Payable** | | | | | | | **4.5 Total of duty payable** | | | | | | | **4.6 Any other payments** | | | | | | |
| Hamle 2005 | \|  \|  \|  \| \| --- \| --- \| --- \| | | |  |  |  |  |  |  |  |  |  |  |  |  |  |  |  |  |  |  |  |  |  |  |  |  |  |  |  |  |  |  |  |  |  |  |  |
| Nahase 2005 | \|  \|  \|  \| \| --- \| --- \| --- \| | | |  |  |  |  |  |  |  |  |  |  |  |  |  |  |  |  |  |  |  |  |  |  |  |  |  |  |  |  |  |  |  |  |  |  |  |
| Meskerem 2006 | \|  \|  \|  \| \| --- \| --- \| --- \| | | |  |  |  |  |  |  |  |  |  |  |  |  |  |  |  |  |  |  |  |  |  |  |  |  |  |  |  |  |  |  |  |  |  |  |  |
| Tikimt 2006 | \|  \|  \|  \| \| --- \| --- \| --- \| | | |  |  |  |  |  |  |  |  |  |  |  |  |  |  |  |  |  |  |  |  |  |  |  |  |  |  |  |  |  |  |  |  |  |  |  |
| Hidar 2006 | \|  \|  \|  \| \| --- \| --- \| --- \| | | |  |  |  |  |  |  |  |  |  |  |  |  |  |  |  |  |  |  |  |  |  |  |  |  |  |  |  |  |  |  |  |  |  |  |  |
| Tahsas 2006 | \|  \|  \|  \| \| --- \| --- \| --- \| | | |  |  |  |  |  |  |  |  |  |  |  |  |  |  |  |  |  |  |  |  |  |  |  |  |  |  |  |  |  |  |  |  |  |  |  |
| Tirr 2006 | \|  \|  \|  \| \| --- \| --- \| --- \| | | |  |  |  |  |  |  |  |  |  |  |  |  |  |  |  |  |  |  |  |  |  |  |  |  |  |  |  |  |  |  |  |  |  |  |  |
| Yekatit 2006 | \|  \|  \|  \| \| --- \| --- \| --- \| | | |  |  |  |  |  |  |  |  |  |  |  |  |  |  |  |  |  |  |  |  |  |  |  |  |  |  |  |  |  |  |  |  |  |  |  |
| Megabit 2006 | \|  \|  \|  \| \| --- \| --- \| --- \| | | |  |  |  |  |  |  |  |  |  |  |  |  |  |  |  |  |  |  |  |  |  |  |  |  |  |  |  |  |  |  |  |  |  |  |  |
| Meyazia 2006 | \|  \|  \|  \| \| --- \| --- \| --- \| | | |  |  |  |  |  |  |  |  |  |  |  |  |  |  |  |  |  |  |  |  |  |  |  |  |  |  |  |  |  |  |  |  |  |  |  |
| Ginbot 2006 | \|  \|  \|  \| \| --- \| --- \| --- \| | | |  |  |  |  |  |  |  |  |  |  |  |  |  |  |  |  |  |  |  |  |  |  |  |  |  |  |  |  |  |  |  |  |  |  |  |
| Sene 2006 | \|  \|  \|  \| \| --- \| --- \| --- \| | | |  |  |  |  |  |  |  |  |  |  |  |  |  |  |  |  |  |  |  |  |  |  |  |  |  |  |  |  |  |  |  |  |  |  |  |
| **Total** |  | | |  |  |  |  |  |  |  |  |  |  |  |  |  |  |  |  |  |  |  |  |  |  |  |  |  |  |  |  |  |  |  |  |  |  |  |

1. **DETERMINING STAFF ALLOCATIONS ACROSS DEPARTMENTS**

**Instructions:**

1. Interview the case team leaders for each major category (doctors, nurses, midwives) of staff. Record the distribution of each category across different departments for the last completed week.
2. For staff which were absent during the last week, ask “***If the person was not absent during the last week, where would that person be working?***”
3. Certain categories of staff, like the medical director and the nursing matron, usually perform clinical as well as administrative duties. Make sure this allocation is captured properly. This may require deeper probing.
4. Total number of staff or man-days (5.1 and 5.12) can only be whole numbers (0, 1, 2, …)
5. 5.2 – 5.11 can be whole numbers or fractions (like 0.5, 1.7, …)
6. For staff categories not available in the health facility, write “0” in the relevant cell of 5.1, and leave other columns blank.

| **ALLOCATION OF TECHNICAL STAFF TO THE HEALTH FACILITY DEPARTMENTS** | | | | | | | | | | | | | |
| --- | --- | --- | --- | --- | --- | --- | --- | --- | --- | --- | --- | --- | --- |
|  | | **Distribution of staffs by department (in Man Days)** | | | | | | | | | | | |
| **Sub-category** | | **5.1 Total Number of Staff** | **5.2**  **OPD** | **5.3**  **ART Clinic** | **5.4**  **MCH** | **5.5**  **Delivery** | **5.6**  **IPD (ward)** | **5.7**  **OR** | **5.8 Management / Admin.** | **5.9 Others 1** | 5.10 Others 2 | 5.11 Others 3 | **5.12**  **Total** |
|  |  |  |  |  |  |  |  |  |  | **a. ________** | **b. ________** | **c. ________** |  |
|  | **DOCTORS** |  |  |  |  |  |  |  |  |  |  |  |  |
| **D1** | **Medical director** |  |  |  |  |  |  |  |  |  |  |  |  |
| D2 | Surgeons |  |  |  |  |  |  |  |  |  |  |  |  |
| D3 | Gynecologists |  |  |  |  |  |  |  |  |  |  |  |  |
| D4 | Pediatricians |  |  |  |  |  |  |  |  |  |  |  |  |
| D5 | Internists |  |  |  |  |  |  |  |  |  |  |  |  |
| D6 | General Practitioners |  |  |  |  |  |  |  |  |  |  |  |  |
| D7 | Emergency Surgical Officers |  |  |  |  |  |  |  |  |  |  |  |  |
| D8 | Others |  |  |  |  |  |  |  |  |  |  |  |  |
|  |  |  |  |  |  |  |  |  |  |  |  |  |  |
|  |  |  |  |  |  |  |  |  |  |  |  |  |  |
|  | **NURSES** |  |  |  |  |  |  |  |  |  |  |  |  |
| N1 | **Nursing matron** |  |  |  |  |  |  |  |  |  |  |  |  |
| N2 | M.Sc. Nurse |  |  |  |  |  |  |  |  |  |  |  |  |
| N3 | B.Sc. Nurse |  |  |  |  |  |  |  |  |  |  |  |  |
| N4 | Diploma nurse |  |  |  |  |  |  |  |  |  |  |  |  |
| N5 | Certificate nurse |  |  |  |  |  |  |  |  |  |  |  |  |
|  |  |  |  |  |  |  |  |  |  |  |  |  |  |
|  |  |  |  |  |  |  |  |  |  |  |  |  |  |
|  | **OTHERS** |  |  |  |  |  |  |  |  |  |  |  |  |
| H1 | Health officers |  |  |  |  |  |  |  |  |  |  |  |  |
| M1 | Midwives |  |  |  |  |  |  |  |  |  |  |  |  |
|  |  |  |  |  |  |  |  |  |  |  |  |  |  |
|  |  |  |  |  |  |  |  |  |  |  |  |  |  |
|  |  |  |  |  |  |  |  |  |  |  |  |  |  |

1. **BUDGET AND EXPENDITURE FOR THE FISCAL YEAR 2006 (*HAMLE 2005 TO SENE 2006*)**

Instructions:

1. This data is for the fiscal year 2006 (*Hamle* 2005 to *Sene* 2006). **There should be no deviations from this rule.**
2. Information for this form is available in the Finance department of the health facility. Ask the department in-charge for **Form Mehi/22** of the standard formats provided by the Regional Finance Bureau. Figures for “Treasury” are sometimes not available at the health center. In such cases, collect the same from the Woreda finance office.
3. These formats are standardized by the Ministry of Finance and Economic Development across the country and sectors. **Please match the “Account code” of the form with that available in the data collection tool carefully before copying the relevant monetary figures.**
4. There are two versions of this form – one for **Treasury**, the other for **Internal Revenue**. Hence, two tables are provided for collecting this information.
5. Most frequently occurring categories are in **bold**.
6. For the cells which are blank in the source document, leave them blank in this form as well.

| **6.1 FROM TREASURY** | | | |
| --- | --- | --- | --- |
| **Account Description** | **Code** | 1. **Adjusted budget** | 1. **Total expenditure year to-date** |
| **Salaries to permanent staff** | **6111** |  |  |
| Salaries of military staff | 6112 |  |  |
| **Wages to contract staff** | **6113** |  |  |
| Wages to casual staff | 6114 |  |  |
| Wages to external contract staff | 6115 |  |  |
| Miscellaneous payments to staff | 6116 |  |  |
| **Allowances to Permanent staff** | **6121** |  |  |
| Allowances to contract staff | 6123 |  |  |
| **Allowances to external contract staff** | **6124** |  |  |
| **Govt contribution to permanent staff pension** | **6131** |  |  |
| Government contribution to military staff pension | 6132 |  |  |
| Uniforms, clothing, bedding | 6211 |  |  |
| Office supplies | 6212 |  |  |
| **Printing** | **6213** |  |  |
| **Medical supplies** | **6214** |  |  |
| Education supplies | 6215 |  |  |
| Food | 6216 |  |  |
| Fuel and lubricants | 6217 |  |  |
| Other materials and supplies | 6218 |  |  |
| Miscellaneous equipments | 6219 |  |  |
| Agriculture, forestry and mine inputs | 6221 |  |  |
| Veterinary supplies and drugs | 6222 |  |  |
| Research and Development supplies | 6223 |  |  |
| Ammunition and ordnance | 6224 |  |  |
| **Per diem** | **6231** |  |  |
| **Transport fees** | **6232** |  |  |
| Official entertainment | 6233 |  |  |
| M/R of vehicles and other transport | 6241 |  |  |
| M/R of aircraft and boats | 6242 |  |  |
| M/R plant, machinery & equipment | 6243 |  |  |
| M/R buildings, furnishing & fixtures | 6244 |  |  |
| M/R of infrastructure | 6245 |  |  |
| Maintenance and repair of military equipment | 6246 |  |  |
| Contracted Professional Services | 6251 |  |  |
| Rent | 6252 |  |  |
| Advertising | 6253 |  |  |
| Insurance | 6254 |  |  |
| Freight | 6255 |  |  |
| Fees and charges | 6256 |  |  |
| Electric charges | 6257 |  |  |
| Telecommunication charges | 6258 |  |  |
| Water and other utilities | 6259 |  |  |
| Local training | 6271 |  |  |
| External training | 6272 |  |  |
| Stocks of food | 6281 |  |  |
| Stocks of fuel | 6282 |  |  |
| Other stocks | 6283 |  |  |
| Purchase of vehicles/other transport | 6311 |  |  |
| Purchase of aircarft, boats, etc. | 6312 |  |  |
| Purchase of plant, machinery & equipment | 6313 |  |  |
| Purchase of buidlings, furnishings & fixtures | 6314 |  |  |
| Purchase of livestock and transport animals | 6315 |  |  |
| Preconstruction activities | 6321 |  |  |
| Construction of buildings - residential | 6322 |  |  |
| Construction of buildings - non residential | 6323 |  |  |
| Construction of infrastructure | 6324 |  |  |
| Supervision of construction works | 6326 |  |  |
| Subsidies, investments and grant payments | 6410 |  |  |
| Grants, contrib. & subsidies to inst & enter | 6412 |  |  |
| Contributions to international organizations | 6414 |  |  |
| Compensations to individuals and institutions | 6416 |  |  |
| Grants and gratitude to individuals | 6417 |  |  |
| Miscellaneous payments | 6419 |  |  |
| **TOTAL** |  |  |  |

| **6.2 FROM INTERNAL REVENUE** | | | |
| --- | --- | --- | --- |
| **Account description** | **Code** | 1. **Adjusted budget** | 1. **Total expenditure year to-date** |
| **Salaries to permanent staff** | **6111** |  |  |
| Salaries of military staff | 6112 |  |  |
| **Wages to contract staff** | **6113** |  |  |
| Wages to casual staff | 6114 |  |  |
| Wages to external contract staff | 6115 |  |  |
| Miscellaneous payments to staff | 6116 |  |  |
| **Allowances to Permanent staff** | **6121** |  |  |
| Allowances to contract staff | 6123 |  |  |
| **Allowances to external contract staff** | **6124** |  |  |
| **Govt contribution to permanent staff pension** | **6131** |  |  |
| Government contribution to military staff pension | 6132 |  |  |
| Uniforms, clothing, bedding | 6211 |  |  |
| Office supplies | 6212 |  |  |
| **Printing** | **6213** |  |  |
| **Medical supplies** | **6214** |  |  |
| Education supplies | 6215 |  |  |
| Food | 6216 |  |  |
| Fuel and lubricants | 6217 |  |  |
| Other materials and supplies | 6218 |  |  |
| Miscellaneous equipments | 6219 |  |  |
| Agriculture, forestry and mine inputs | 6221 |  |  |
| Veterinary supplies and drugs | 6222 |  |  |
| Research and Development supplies | 6223 |  |  |
| Ammunition and ordnance | 6224 |  |  |
| **Per diem** | **6231** |  |  |
| **Transport fees** | **6232** |  |  |
| Official entertainment | 6233 |  |  |
| M/R of vehicles and other transport | 6241 |  |  |
| M/R of aircraft and boats | 6242 |  |  |
| M/R plant, machinery & equipment | 6243 |  |  |
| M/R buildings, furnishing & fixtures | 6244 |  |  |
| M/R of infrastructure | 6245 |  |  |
| Maintenance and repair of military equipment | 6246 |  |  |
| Contracted Professional Services | 6251 |  |  |
| Rent | 6252 |  |  |
| Advertising | 6253 |  |  |
| Insurance | 6254 |  |  |
| Freight | 6255 |  |  |
| Fees and charges | 6256 |  |  |
| Electric charges | 6257 |  |  |
| Telecommunication charges | 6258 |  |  |
| Water and other utilities | 6259 |  |  |
| Local training | 6271 |  |  |
| External training | 6272 |  |  |
| Stocks of food | 6281 |  |  |
| Stocks of fuel | 6282 |  |  |
| Other stocks | 6283 |  |  |
| Purchase of vehicles/other transport | 6311 |  |  |
| Purchase of aircarft, boats, etc. | 6312 |  |  |
| Purchase of plant, machinery & equipment | 6313 |  |  |
| Purchase of buidlings, furnishings & fixtures | 6314 |  |  |
| Purchase of livestock and transport animals | 6315 |  |  |
| Preconstruction activities | 6321 |  |  |
| Construction of buildings - residential | 6322 |  |  |
| Construction of buildings - non residential | 6323 |  |  |
| Construction of infrastructure | 6324 |  |  |
| Supervision of construction works | 6326 |  |  |
| Subsidies, investments and grant payments | 6410 |  |  |
| Grants, contrib. & subsidies to inst & enter | 6412 |  |  |
| Contributions to international organizations | 6414 |  |  |
| Compensations to individuals and institutions | 6416 |  |  |
| Grants and gratitude to individuals | 6417 |  |  |
| Miscellaneous payments | 6419 |  |  |
| **TOTAL** |  |  |  |

1. **REVENUE FOR THE FISCAL YEAR 2006 (HAMLE 2005 TO SENE 2006)**

**Instructions for Table 7.1 and 7.2:**

1. All figures in this section are to be recorded for the fiscal year 2006 (Hamel 2005 to Sene 2006).
2. Information on ***Table 7.1*** is available from Form Mehi/21 provided by the Regional Finance Bureau. It is also called “Revenue (Assistance) Loan Report”.
3. ***Table 7.1*** has standard codes and account descriptions for frequently occurring revenue sources. Match these codes between the source document and this form carefully. If other revenue codes are mentioned in the source document please use the blank rows to record the “Account description”, the “Code” and the total revenue figure.
4. ***Table 7.2***: Information for **Fee waiver** is available from the request letters written by the facility head to the Woreda or Regional administration (or health insurance scheme). This letter should be available with the Finance department or the facility-in-charge, or Woreda finance office.
5. ***Table 7.2***: Information for **Community contributions** and **Other Donations** can be obtained from the facility in charge. This information might be recorded in official documents. If not, interview the facility in charge to obtain estimates of these figures.
6. ***Table 7.2***: **Other Donations** do not include program drugs and supplies (which is being estimated elsewhere)
7. For “**Other donations**” that are being sent from the Woredas, ask the facility in charge to estimate the value of the items received. In cases where the facility in charge is unable to estimate a value, contact the Woreda office for this purpose.

Table 7.1

| **S. No.** | **Account description** | **Code** | **Total revenue year to-date** |
| --- | --- | --- | --- |
| 7.1.1 | Drugs and supplies | **1436** |  |
| 7.1.2 | Health care services (Registration card, Laboratory fee) | **1437** |  |
| 7.1.3 | Other miscellaneous revenue | **1489** |  |
| 7.1.4 |  |  |  |
| 7.1.5 |  |  |  |
| 7.1.6 |  |  |  |

Table 7.2

| **S.No.** | **Source of revenue** | **Number / Amount (In Birr)** |
| --- | --- | --- |
| **7.2.1** | **Fee waiver information** |  |
| 7.2.1.1 | Total **number** of fee waived patients |  |
| 7.2.1.1.1 | Total **number** of waived patients (**outpatient**) |  |
| 7.2.1.1.2 | Total **number** of waived patients (**inpatient**) |  |
|  |  |  |
| 7.2.1.2 | Total **amount** of waived fees |  |
| 7.2.1.3 | Total **amount** of waived fees reimbursed from the Woreda / region |  |
| 7.2.1.4 | Interviewer: Ask the following question from the in-charge of the finance department:  *Is the total amount of waived fees reimbursed from the Woreda / region, a part of the revenue reported in Mehi/21?*  ***Yes = 01, No=02***  **Interviewer: The answer to this question should usually be “Yes”. If it’s “No” please ask for the reason behind this practice and record the reason in the comment box of this form (with question number).** | \|  \|  \| \| --- \| --- \| |
| 7.2.1.5 | Interviewer: If response to the previous question is “YES”, ask this follow-up question:  “*Under which standard code, is this revenue included?”*  Record the standard code(s) in the boxes provided. | \|  \|  \|  \|  \| \| --- \| --- \| --- \| --- \|  \|  \|  \|  \|  \| \| --- \| --- \| --- \| --- \|  \|  \|  \|  \|  \| \| --- \| --- \| --- \| --- \| |
| 7.2.2 | Community contribution |  |
| 7.2.2.1 | Total contribution |  |
| 7.2.2.1.1 | Contribution in-cash |  |
| 7.2.2.1.2 | Contribution in-kind |  |
|  |  |  |
| 7.2.3 | Other Donations (Excluding program drugs and supplies) |  |
| 7.2.3.1 | Total amount of donation |  |
| 7.2.3.1.1 | Donation in-cash |  |
| 7.2.3.1.2 | Donation in-kind |  |

1. **EPIDEMIOLOGICAL PROFILE**

**Instructions:**

1. Information for this section is usually available on the wall charts pasted in the office of facility in-charge, HMIS office, etc.
2. If no such wall charts are found, interview the HMIS officer for collecting this information
3. Make sure to collect the TOTAL NUMBERS (last row of each table)

| **8.1 Top ten causes of OPD Visit (put actual number not percentages)** | | |  |
| --- | --- | --- | --- |
| **No** | **Disease/Condition** | **Total Number** |  |
| 1 |  |  |  |
| 2 |  |  |  |
| 3 |  |  |  |
| 4 |  |  |  |
| 5 |  |  |  |
| 6 |  |  |  |
| 7 |  |  |  |
| 8 |  |  |  |
| 9 |  |  |  |
| 10 |  |  |  |
|  | Others |  |  |
|  | **Total** |  |  |
|  |  |  |  |
| 8.2 Top ten causes of IPD (put actual number not percentages) | | |  |
| **No** | **Disease/Condition** | **Total Number** |  |
| 1 |  |  |  |
| 2 |  |  |  |
| 3 |  |  |  |
| 4 |  |  |  |
| 5 |  |  |  |
| 6 |  |  |  |
| 7 |  |  |  |
| 8 |  |  |  |
| 9 |  |  |  |
| 10 |  |  |  |
|  | Others |  |  |
|  | **Total** |  |  |

1. **SERVICE STATISTICS**

**9a. Information from Health Management Information System (HMIS)**

**Instructions:**

1. The source of this information is the Health Management Information System (HMIS). This data can be found stored in the electronic format (e-HMIS), and/or as printed forms in the health facility. **Do not use other sources such as the Key Performance Indicators (KPI) for this section.**
2. For the F.Y. 2006, this data is maintained in the form of quarterly and/or monthly reports. Preference should be given to have the four quarterly reports which represent the whole F.Y. 2006. Please add up the numbers for each field from the four quarterly reports to arrive at the final figure for the whole year, and record that figure in front of the relevant field.
3. In case the quarterly reports are not available, collect the monthly reports for the whole year and add them up to arrive at the annual figure.
4. Tracer drug availability will show 0 or 1 value for each month; count the number of months with a value 1 and enter the total (if a facility has value 1 in 8 of the 12 months for a particular tracer drug then the tracer availability for that particular drug will be 8)
5. **Do not leave any field blank in this section.** If the data is not available for a particular field for the completed F.Y. 2006, contact the survey coordinators / Addis office immediately. If you are not able to contact them, mention “missing” for the particular field of data and write a detailed reason in the comments box of this form. Never write “0” or any other number in a field with missing data. Do not attempt to guess the annual figure from incomplete information.

| **S. No.** | **Data Element** | **Total number (Year 2006)** |
| --- | --- | --- |
|  | Family Planning Acceptors |  |
| **1** | **Total new and repeat acceptors** |  |
| 2 | New acceptors |  |
| 3 | Repeat acceptors |  |
|  | Antenatal Care |  |
| 4 | First antenatal care attendances |  |
| 5 | Total number of antenatal care visits (New and repeat) |  |
|  | Abortion care |  |
| 6 | Abortions (Number of abortions conducted by the health facility) |  |
|  | Deliveries and Outcomes |  |
| 7 | Number of deliveries attended by skilled attendant |  |
| 8 | Number of caesarean sections performed |  |
|  | Postnatal Care |  |
| 9 | First post natal attendances |  |
| 10 | Total number of post natal attendances |  |
|  | Child Health |  |
|  | Expanded Program on Immunization (EPI) |  |
| 11 | Pentavalent DPT1-HepB1-Hib1 immunizations for infants < 1 year of age |  |
| 12 | Pentavalent DPT3-HepB3-Hib3 immunizations for infants < 1 year of age |  |
| 13 | Pneumococcal 1 immunization for infants < 1year of age |  |
| 14 | Pneumococcal 2 immunization for infants < 1year of age |  |
| 15 | Pneumococcal 3 immunization for infants < 1year of age |  |
| 16 | Rota 1 immunization for infants < 1year of age |  |
| 17 | Rota 2 immunization for infants < 1year of age |  |
| 18 | Measles immunizations for infants < 1 year of age |  |
| 19 | Fully immunized infants < 1 year of age |  |
| 20 | Births protected against NNT (PAB) |  |
|  | Vaccine |  |
| 21 | BCG doses given (all ages) |  |
| 22 | BCG doses opened |  |
| 23 | Pentavalent (DPT-HepB-Hib) doses given (all ages) |  |
| 24 | Pentavalent (DPT-HepB-Hib) doses opened |  |
| 25 | Polio doses given (all ages) |  |
| 26 | Polio doses opened |  |
| 27 | Measles doses given (all ages) |  |
| 28 | Measles doses opened |  |
| 29 | Pneumococcal doses given (all age) |  |
| 30 | Pneumococcal doses opened |  |
| 31 | Rota doses given (all ages) |  |
| 32 | Rota doses opened |  |
| 33 | TT doses given (all ages) |  |
| 34 | TT doses opened |  |
|  | Disease Prevention and Control |  |
|  | TB and Leprosy |  |
|  | New TB cases detected by TB DOTS program |  |
| 35 | Number of new smear positive pulmonary TB cases detected by TB DOTS program |  |
| 36 | Number of new smear negative pulmonary TB cases detected by TB DOTS program |  |
| 37 | Number of new extra-pulmonary TB cases detected by TB DOTS program |  |
|  | **Results for pulmonary TB cohort completing this quarter** |  |
| 37 | Smear positive TB cases enrolled in cohort completing this quarter |  |
| 38 | Treatment completed PTB+ |  |
| 39 | Cured PTB+ |  |
|  | **Leprosy cases and treatment results** |  |
| 40 | Leprosy (new cases) (MB+PB) |  |
| 41 | New leprosy cases under 15 |  |
|  | **Leprosy cases and treatment results** |  |
| 42 | Leprosy (new cases) (MB+PB) |  |
| 43 | New leprosy cases under 15 |  |
| 44 | Treatment completed leprosy: PB |  |
|  | TB/HIV Coinfection |  |
| 45 | Number of TB patients enrolled in DOTS and tested for HIV in this quarter |  |
| 46 | Number of TB patients enrolled in DOTS who are HIV + in this quarter |  |
|  | **HIV/AIDS** |  |
|  | ART |  |
| 47 | Number of PLWHA ever enrolled in HIV care |  |
| 48 | Number of PLWHA ever started on ART |  |
| 49 | Number of PLWHA currently receiving ART |  |
|  | Adults |  |
| 50 | Adult First Line Regimen |  |
| 51 | Adult Second Line Regimen |  |
|  | Children |  |
| 52 | Child First Line Regimen |  |
| 53 | Child Second Line Regimen |  |
|  | Logistics |  |
|  | Tracer drug availability (enter 1 if drug available whenever needed, 0 if ever unavailable when needed). |  |
| 54 | Amoxicillin |  |
| 55 | Oral Rehydration Salt |  |
| 56 | Arthemisin / Lumphantrine |  |
| 57 | Mebendazole Tablets |  |
| 58 | Tetracycline Eye Ointment |  |
| 59 | Paracetamol |  |
| 60 | Refampicine / Isoniazide / Pyrazinamide / Ethambutol |  |
| 61 | Medroxyprogesterone (depo) Injection |  |
| 62 | Ergometrine Maleate Tablets |  |
| 63 | Ferrous Salt plus Folic Acid |  |
| 64 | Pentavalent DPT-Hep-Hib Vaccine |  |
| 65 | Months x tracer drugs |  |
|  | Health Systems |  |
|  | Health service coverage and utilization |  |
| 66 | OPD visits < 5: new Male |  |
| 67 | OPD visits < 5: new Female |  |
| 68 | OPD visits < 5: repeat Male |  |
| 69 | OPD visits < 5: repeat Female |  |
| 70 | OPD visits 5-14: new Male |  |
| 71 | OPD visits 5-14: new Female |  |
| 72 | OPD visits 5-14: repeat Male |  |
| 73 | OPD visits 5-14: repeat Female |  |
| 74 | OPD visits >= 15: new Male |  |
| 75 | OPD visits >= 15: new Female |  |
| 76 | OPD visits >= 15: repeat Male |  |
| 77 | OPD visits >= 15: repeat Female |  |
| 78 | Number of practitioners working in OPD |  |
| 79 | Number of admissions |  |
| 80 | Total length of stay (in days) |  |
| 81 | Number of beds |  |
| 82 | Number of discharges |  |
|  | HMIS and M&E |  |
| 83 | Data quality LQAS score |  |
|  | Malaria |  |
| 84 | Number of new malaria cases |  |

**9b. Information on Child Health and Nutrition**

Information for this section is usually available in the form of program reports available at the health facility. These reports can be monthly, quarterly, or annual. Make sure that data for this section is collected for entire EFY 2006.

| 1. **Integrated Community Case Management of Childhood Illnesses (ICCM)** | | |
| --- | --- | --- |
| 1.1 | Interviewer: Ask the following question from the facility in-charge:  *Were ICCM services provided at this health facility at any point of time during the EFY 2006?*  ***Yes = 01, No=02*** | \|  \|  \| \| --- \| --- \| |
| 1.2 | Interviewer: If 1.1 is “01”, ask the facility in-charge for the total number of ICCM cases treated by the Health Extension Workers (HEW) of this health facility, during EFY 2006. Record the number in the space provided in the right column.  This information is usually recorded and reported in the program statistics report of ICCM. |  |

| 1. **Integrated Management of Childhood Illnesses (IMCI)** | | |
| --- | --- | --- |
| 2.1 | Interviewer: Ask the following question from the facility in-charge:  *Were IMCI services provided at this health facility at any point of time during the EFY 2006?*  ***Yes = 01, No=02*** | \|  \|  \| \| --- \| --- \| |
| 2.2 | Interviewer: If 2.1 is “01”, ask the facility in-charge for the total number of IMCI cases treated at this health facility during EFY 2006. Record the number in the space provided in the right column.  This information is usually recorded and reported in the program statistics report of IMCI. |  |

| 1. **Community Management of Acute Malnutrition (CMAM)** | | |
| --- | --- | --- |
| 3.1 | Interviewer: Ask the following question from the facility in-charge:  *Were CMAM services provided at this health facility at any point of time during the EFY 2006?*  ***Yes = 01, No=02*** | \|  \|  \| \| --- \| --- \| |
| 3.2 | Interviewer: If 3.1 is “01”, ask the facility in-charge for the total number of CMAM cases treated at this health facility during EFY 2006. Record the number in the space provided in the right column.  This information is usually recorded and reported in the program statistics report of CMAM. |  |

**9c. Voluntary Counseling and Testing (VCT) services**

Information for this section is usually available in the form of program reports available at the VCT center of health facility. These reports can be monthly, quarterly, or annual. Make sure that data for this section is collected for entire EFY 2006. Contact the facility in charge or the case team leader of VCT services to access these reports.

| 1. **Voluntary Counseling and Testing (VCT) services** | | |
| --- | --- | --- |
| 1.1 | Interviewer: Ask the following question from the facility in-charge:  *Were VCT services provided at this health facility at any point of time during the EFY 2006?*  ***Yes = 01, No=02*** | \|  \|  \| \| --- \| --- \| |
| 1.2 | Interviewer: If 1.1 is “01”, ask the facility in-charge for the total number of clients at the health facility during EFY 2006 for the following categories:   1. Total number of clients which received pre-test counseling 2. Total number of clients which received HIV testing 3. Total number of clients which tested positive for HIV | \|  \| \| --- \|  \|  \| \| --- \|  \|  \| \| --- \| |
|  | 1. Total number of clients which received PIHCT counseling 2. Total number of PIHCT clients which received HIV testing 3. Total number of PIHCT clients which tested positive for HIV | \|  \| \| --- \|  \|  \| \| --- \|  \|  \| \| --- \| |

1. **DRUGS AND SUPPLIES**

**Section 1: Quantity of program drugs and supplies consumed during the F.Y. 2006**

Instructions:

1. This section has to be completed with the person in-charge of the drug store or his/her representative.
2. Information for this section comes from the Report and Requisition Forms (RRFs) which follow a standardized format.
3. Three different types of RRFs should be requested from each health center:

Table 10.1 applies to the Program Drugs only.
Table 10.2 applies to Program reagents, supplies and diagnostics for non-lab monitoring health centers

Table 10.3 applies to Program reagents, supplies and diagnostics for lab monitoring health centers

*RRF for Table 10.1 is found in* ***all*** *health centers.*

*However, amongst Tables 10.2 and Table 10.3, certain health centers would have RRFs for only one of them. Ask for the reason of the missing RRF and record in the comments box of this form. Health facilities often use their own formats to report subjects on Table 10.2 and Table 10.3 copy the data from their report to the tables.*

1. The RRFs, usually, are prepared every two months, which means there should be a total of 6 RRFs for the full F.Y. 2006. So, normally only the first six columns would be used, but 3 extra columns have been provided to accommodate deviations from this practice.
2. Check the units in the RRF (Tables 10.1 – 10.3) exactly match with the RRF in the facility report. Where the units are empty in the tables fill according to what is reported in the facility report.
3. Health Facilities are reporting in “odd” or “even” number months (following E.C); in the case of “even” number reporting health facilities the starting period will be Sene 2005 and the ending period will be Ginbot 2006.
4. **Column E of the RRFs is the calculated consumption which has to be recorded** for each drug for all the RRFs that comprise of F.Y. 2006.
5. There should be no negative value in the consumption column (Column E), if you find negative values it is a mistake and should be noted in the comment box.
6. Record in only those fields where a non-zero number is present. Match the name of drugs in the tables with the list in RRFs carefully, as different versions of RRFs place drugs in different order (although nearby).

**TABLE 10.1 PROGRAM DRUGS**

| **Period** | | | **1** | **2** | **3** | **4** | **5** | **6** | **7** | **8** | **9** |
| --- | --- | --- | --- | --- | --- | --- | --- | --- | --- | --- | --- |
| **BEGINNING DATE🡪** | | |  |  |  |  |  |  |  |  |  |
| **ENDING DATE🡪** | | |  |  |  |  |  |  |  |  |  |
| **Ser. No.** | **Product description** | **Unit of issue** |  |  |  |  |  |  |  |  |  |
|  | 1. **ARVs (HPCP I)** |  |  |  |  |  |  |  |  |  |  |
| 1 | Abacavir 20mg/ml, 240ml susp | Bottle |  |  |  |  |  |  |  |  |  |
| 2 | Abacavir 300mg, 60 tabs | Pk |  |  |  |  |  |  |  |  |  |
| 3 | Atazanavir – Ritonavir 300+100 mg, 30 tabs | Pk |  |  |  |  |  |  |  |  |  |
| 4 | Atazanavir – Ritonavir 150+80 mg, 30 tabs | Pk |  |  |  |  |  |  |  |  |  |
| 5 | Didanosine 2g powder | Bottle |  |  |  |  |  |  |  |  |  |
| 6 | Efavirenz 30mg/ml, 180ml solution | Bottle |  |  |  |  |  |  |  |  |  |
| 7 | Efaviernz 50mg, 30 caps | Pk |  |  |  |  |  |  |  |  |  |
| 8 | Efaviernz 200mg, 90 caps | Pk |  |  |  |  |  |  |  |  |  |
| 9 | Efaviernz 600mg, 30 caps | Pk |  |  |  |  |  |  |  |  |  |
| 10 | Lamivudine 10mg/ml, 240ml solution | Bottle |  |  |  |  |  |  |  |  |  |
| 11 | Lamivudine 150mg, 60 tabs | Pk |  |  |  |  |  |  |  |  |  |
| 12 | Lamivudine 300mg/Tenofovir 300mg, 30 tabs | Pk |  |  |  |  |  |  |  |  |  |
| 13 | Lamivudine 30mg/Zidovudine 60mg, 60 tabs | Pk |  |  |  |  |  |  |  |  |  |
| 14 | Lamivudine 30mg/ Zidovudine 60mg/Nevirapine 50mg, 60 tabs | Pk |  |  |  |  |  |  |  |  |  |
| 15 | Lopinavir/Ritonvavir, 80mg/20mg/ml, 60ml solution | Bottle |  |  |  |  |  |  |  |  |  |
| 16 | Nevirapine 10mg/ml, 20ml susp | Bottle |  |  |  |  |  |  |  |  |  |
| 17 | Nevirapine 10mg/ml, 240ml susp | Bottle |  |  |  |  |  |  |  |  |  |
| 18 | Nevirapine 200mg, 60 tabs | Pk |  |  |  |  |  |  |  |  |  |
| 19 | Stavudine 12mg/Lamivudine 60mg, 60 tabs | Pk |  |  |  |  |  |  |  |  |  |
| 20 | Stavudine 12mg/Lamivudine 60mg/Nevirapine 100 mg, 60 tabs | Pk |  |  |  |  |  |  |  |  |  |
| 21 | Stavudine 1mg/ml, 200ml susp | Pk |  |  |  |  |  |  |  |  |  |
| 22 | Stavudine 15mg, 60 caps | Bottle |  |  |  |  |  |  |  |  |  |
| 23 | Stavudine 20mg, 60 caps | Pk |  |  |  |  |  |  |  |  |  |
| 24 | Stavudine 6mg/Lamivudine 30 mg, 60 tabs | Pk |  |  |  |  |  |  |  |  |  |
| 25 | Stavudine 30mg/Lamivudine 150 mg, 60 tabs | Pk |  |  |  |  |  |  |  |  |  |
| 26 | Stavudine 6mg/Lamivudine 30 mg/Nevirapine 50mg, 60 tabs | Pk |  |  |  |  |  |  |  |  |  |
| 27 | Stavudine 30mg/Lamivudine 150mg/Nevirapine 200mg, 60 tabs | Pk |  |  |  |  |  |  |  |  |  |
| 28 | Tenofovir 300mg, 30 tabs | Pk |  |  |  |  |  |  |  |  |  |
| 29 | Tenofovir 300mg,Emtricitabine 200mg/Efavirenz 600mg, 30 tabs | Pk |  |  |  |  |  |  |  |  |  |
| 30 | Zidovudine 10mg/ml, 100ml susp | Pk |  |  |  |  |  |  |  |  |  |
| 31 | Zidovudine 10mg/ml, 240ml susp | Bottle |  |  |  |  |  |  |  |  |  |
| 32 | Zidovudine 100mg, 100 caps | Bottle |  |  |  |  |  |  |  |  |  |
| 33 | Zidovudine 300mg, 60 caps | Pk |  |  |  |  |  |  |  |  |  |
| 34 | Zidovudine 300mg/Lamivudine 150 mg, 60 tabs | Pk |  |  |  |  |  |  |  |  |  |
| 35 | Zidovudine 300mg/Lamivudine 150 mg/Nevirapine 200 mg, 60 tabs | Pk |  |  |  |  |  |  |  |  |  |
| 36 |  |  |  |  |  |  |  |  |  |  |  |
| 37 |  |  |  |  |  |  |  |  |  |  |  |
| 38 |  |  |  |  |  |  |  |  |  |  |  |
|  | **B. Food-By-Prescription (HPCP II)** |  |  |  |  |  |  |  |  |  |  |
| 39 | F100 (Formula 100) 456gm | Pack |  |  |  |  |  |  |  |  |  |
| 40 | F75 (Formula 75) 410gm | Pack |  |  |  |  |  |  |  |  |  |
| 41 | Food By Prescription (Corn Soya Milk Blended) 100gm | Sachet |  |  |  |  |  |  |  |  |  |
| 42 | Food By Prescription (Corn Soya Milk Blended) 200gm | Sachet |  |  |  |  |  |  |  |  |  |
| 43 | Ready To Use Therapeutic Food (Plumpy Nut) 92gm | Sachet |  |  |  |  |  |  |  |  |  |
| 44 | Ready- to-Use Supplementary Food (RUSF) 92gm | Sachet |  |  |  |  |  |  |  |  |  |
|  | **C. Anti Tuberculosis Drugs (TBLPCP I)** |  |  |  |  |  |  |  |  |  |  |
| 45 | Ethambutol 400mg + Isoniazid 150mg, film coated 28 tabs | Pk |  |  |  |  |  |  |  |  |  |
| 46 | Ethambutol HCl 100mg, film coated, 28 tabs | Pk |  |  |  |  |  |  |  |  |  |
| 47 | Isoniazid 100mg tab | Tab |  |  |  |  |  |  |  |  |  |
| 48 | Isoniazid 300mg tab | Tab |  |  |  |  |  |  |  |  |  |
| 49 | Rifampicin 150mg/Isoniazid 75mg/Pyrazinamide 400mg/Ethambutol 275mg, film coated, 28 tabs | Pk |  |  |  |  |  |  |  |  |  |
| 50 | Rifampicin 150mg/Isoniazid 75mg/Pyrazinamide 400mg, film coated, 28 tabs | Pk |  |  |  |  |  |  |  |  |  |
| 51 | Rifampicin 150mg/Isoniazid 75mg, film coated, 28 tabs | Pk |  |  |  |  |  |  |  |  |  |
| 52 | Rifampicin 60mg/Isoniazid 30mg/Pyrazinamide 150mg, film coated, 28 tabs | Pk |  |  |  |  |  |  |  |  |  |
| 53 | Rifampicin 60mg/Isoniazid 30mg, 28tabs | Pk |  |  |  |  |  |  |  |  |  |
| 54 | Streptomycin (as sulphate) 1g, powder for inj. | Vial |  |  |  |  |  |  |  |  |  |
|  | **D. Anti-Leprotics Drugs (TBLPCP I)** |  |  |  |  |  |  |  |  |  |  |
| 55 | **(PB Child)** Rifampicin 400mg (1 cap) + Dapsone 50mg, 28 tabs | Blister |  |  |  |  |  |  |  |  |  |
| 56 | **(PB Adult)** Rifampicin 600mg (1 cap) + Dapsone 100mg, 28 tabs | Blister |  |  |  |  |  |  |  |  |  |
| 57 | (**MB** **Child)** Rifampicin 450mg (1 cap) + Dapsone 50mg (30 tabs) + Clofazimine 100mg (1tab) | Blister |  |  |  |  |  |  |  |  |  |
| 58 | **(MB Adult)** Rifampicin 600mg (1 cap) + Dapson 100mg (30tabs) + Clofazimine 300mg (1 tab) | Blister |  |  |  |  |  |  |  |  |  |
|  | **E. Family Health (FH) Commodities (FHP)** | Pk |  |  |  |  |  |  |  |  |  |
| 59 | Condom (male), 144 Pcs | Pk |  |  |  |  |  |  |  |  |  |
| 60 | Etonogestril – Implant – 68mg (**Implanon**), 64 sets | Pk |  |  |  |  |  |  |  |  |  |
| 61 | IUCD, 50 Sets | Pk |  |  |  |  |  |  |  |  |  |
| 62 | Levornogestrel – Implant – 75mg (**Jadelle**), 10 Sets | Pk |  |  |  |  |  |  |  |  |  |
| 63 | Levornogestrel (D-Norgestrel) + Ethinylestradiol and Iron – Tablet – 0.15mg + 0.03mg (**Microgynon**), 30 Cycles | Pk |  |  |  |  |  |  |  |  |  |
| 64 | Levornogestrel (D-Norgestrel)-Tablet, 0.75mg (**ECP**), strip of 2 | Pk |  |  |  |  |  |  |  |  |  |
| 65 | Levornogestrel (D-Norgestrel)- Tablet, 0.03mg (**Mini Pills**), 30 cycles | Pk |  |  |  |  |  |  |  |  |  |
| 66 | Medroxy progesterone Acetate – Injection 150mg/ml in 1ml vial (**Depo Provera**), 25 Vial | Pk |  |  |  |  |  |  |  |  |  |
|  | **F. Antimalaria Drugs (MPCP)** |  |  |  |  |  |  |  |  |  |  |
| 67 | Arthemether + Lumefanthrine – Tablet – 20mg+120mg (6 X 30) | Pk |  |  |  |  |  |  |  |  |  |
| 68 | Arthemether + Lumefanthrine – Tablet – 20mg+120mg (12 X 30) | Pk |  |  |  |  |  |  |  |  |  |
| 69 | Arthemether + Lumefanthrine – Tablet – 20mg+120mg (18 X 30) | Pk |  |  |  |  |  |  |  |  |  |
| 70 | Arthemether + Lumefanthrine – Tablet – 20mg+120mg (24 X 30) | Pk |  |  |  |  |  |  |  |  |  |
| 71 | Chloroquine Phosphate – Syrup – 50mg/5ml | Bottle |  |  |  |  |  |  |  |  |  |
| 72 | Chloroquine Phosphate 150mg, 1000 tabs | Pk |  |  |  |  |  |  |  |  |  |
| 73 | Quinine Dihydrochloride 300mg, 100 tabs | Pk |  |  |  |  |  |  |  |  |  |
| 74 | Quinine Dihydrochloride – Injection – 300mg/mg in 2ml ampoule, 10 amps | Pk |  |  |  |  |  |  |  |  |  |
| 75 | Acyclovir 200mg, 100 tabs | Pk |  |  |  |  |  |  |  |  |  |
| 76 | Acyclovir 200mg/5ml, 125 susp | Bottle |  |  |  |  |  |  |  |  |  |
| 77 | Acyclovir 400mg, 100 tabs | Pk |  |  |  |  |  |  |  |  |  |
| 78 | Acyclovir 3% eye ointment, 4.5gm | Tube |  |  |  |  |  |  |  |  |  |
| 79 | Albendazole 100mg/5ml, 20ml susp | Bottle |  |  |  |  |  |  |  |  |  |
| 80 | Albendazole 200mg, 100 tabs | Pk |  |  |  |  |  |  |  |  |  |
| 81 | Albendazole 400mg, 100 tabs | Pk |  |  |  |  |  |  |  |  |  |
| 82 | Amoxicillin 125mg/5ml, 100ml susp | Bottle |  |  |  |  |  |  |  |  |  |
| 83 | Amoxicillin 250mg, 1000 caps | Pk |  |  |  |  |  |  |  |  |  |
| 84 | Amoxicillin 250mg/5ml, 100ml susp | Bottle |  |  |  |  |  |  |  |  |  |
| 85 | Amoxicillin 500mg, 500 caps | Pk |  |  |  |  |  |  |  |  |  |
| 86 | Amoxicillin + Clavulinic Acid 125mg+31.25mg/5ml, 100ml susp | Bottle |  |  |  |  |  |  |  |  |  |
| 87 | Amoxicillin + Clavulinic Acid 250mg+62mg/5ml susp, 100ml | Bottle |  |  |  |  |  |  |  |  |  |
| 88 | Amoxicillin 500mg + Clavulinic acid 125mg, 14 tabs | Pk |  |  |  |  |  |  |  |  |  |
| 89 | Amytryptiline 25mg, 100 tabs | Pk |  |  |  |  |  |  |  |  |  |
| 90 | Benzyl benzoate 250mg/5ml, 190ml lotion | Bottle |  |  |  |  |  |  |  |  |  |
| 91 | Benzyl penicillin sodium inj. 10MIU | Vial |  |  |  |  |  |  |  |  |  |
| 92 | Calcium folinate 15mg, 10 tabs | Pk |  |  |  |  |  |  |  |  |  |
| 93 | Carbamazepine 200mg, 1000 tabs | Pk |  |  |  |  |  |  |  |  |  |
| 94 | Cetrizine 5mg, 100 tabs | Pk |  |  |  |  |  |  |  |  |  |
| 95 | Chloramphenicol 1g inj. | Vial |  |  |  |  |  |  |  |  |  |
| 96 | Chlorpheniramine maleate 2mg/5ml syrup, 100ml | Bottle |  |  |  |  |  |  |  |  |  |
| 97 | Chlorpheniramine maleate 4mg, 100 tabs | Pk |  |  |  |  |  |  |  |  |  |
| 98 | Clotrimazole 20g, 1% cream | Tube |  |  |  |  |  |  |  |  |  |
| 99 | Clotrimazole 100mg, 6 vag. tabs | Pk |  |  |  |  |  |  |  |  |  |
| 100 | Clotrimazole 200mg, 3 vag. tabs | Pk |  |  |  |  |  |  |  |  |  |
| 101 | Cloxacillin sodium 125mg/5ml, 100ml susp | Bottle |  |  |  |  |  |  |  |  |  |
| 102 | Cloxacillin sodium 250mg, 1000 caps | Pk |  |  |  |  |  |  |  |  |  |
| 103 | Cloxacillin sodium 500mg, 500 caps | Pk |  |  |  |  |  |  |  |  |  |
| 104 | Dapsone 50mg, 100 tabs | Pk |  |  |  |  |  |  |  |  |  |
| 105 | Dapsone 100mg, 28 tabs | Pk |  |  |  |  |  |  |  |  |  |
| 106 | Dextromethorphan HCl syrup 7.5mg/ml, 100ml syrup | Bottle |  |  |  |  |  |  |  |  |  |
| 107 | Dextrose in normal saline 0.05mg+0.009mg/ml IV infusion, 1lit. | Bag |  |  |  |  |  |  |  |  |  |
| 108 | Diazepam 5mg, 10 tabs | Pk |  |  |  |  |  |  |  |  |  |
| 109 | Diazepam 5mg/ml inj. | Amp |  |  |  |  |  |  |  |  |  |
| 110 | Diclofenac 25mg, 100 tabs | Pk |  |  |  |  |  |  |  |  |  |
| 111 | Diclofenac 50mg, 100 tabs | Pk |  |  |  |  |  |  |  |  |  |
| 112 | Diclofenac 75mg/3ml | Amp |  |  |  |  |  |  |  |  |  |
| 113 | Doxycycline HCl 100mg, 100 caps | Pk |  |  |  |  |  |  |  |  |  |
| 114 | Erythromycin 125mg/5ml, 100ml susp | Bottle |  |  |  |  |  |  |  |  |  |
| 115 | Erythromycin 250mg, 1000 tabs | Pk |  |  |  |  |  |  |  |  |  |
| 116 | Erythromycin 500mg, 100 tabs | Pk |  |  |  |  |  |  |  |  |  |
| 117 | Fluconazole 50mg/5ml, 35ml susp | Bottle |  |  |  |  |  |  |  |  |  |
| 118 | Fluconazole 100mg, 10 tabs | Pk |  |  |  |  |  |  |  |  |  |
| 119 | Fluconazole 200mg, 28 tabs | Pk |  |  |  |  |  |  |  |  |  |
| 120 | Folinic acid 5mg, 10 tabs | Pk |  |  |  |  |  |  |  |  |  |
| 121 | Gentamicin sulfate 80mg inj. | Amp |  |  |  |  |  |  |  |  |  |
| 122 | Hydrocortisone acetate 15gm, 1% cream | Tube |  |  |  |  |  |  |  |  |  |
| 123 |  |  |  |  |  |  |  |  |  |  |  |
|  | **F. OI Drugs II (HPCP III)** |  |  |  |  |  |  |  |  |  |  |
| 124 | Metronidazole 125mg/100ml susp, 30ml | Bottle |  |  |  |  |  |  |  |  |  |
| 125 | Metronidazole 250mg, 1000 caps | Pk |  |  |  |  |  |  |  |  |  |
| 126 | Miconazole 10mg, 70 tabs | Pk |  |  |  |  |  |  |  |  |  |
| 127 | Miconazole 20mg, oral gel | Tube |  |  |  |  |  |  |  |  |  |
| 128 | Miconazole 800mg, gel | Tube |  |  |  |  |  |  |  |  |  |
| 129 | Multivitamin syrup, 100ml | Bottle |  |  |  |  |  |  |  |  |  |
| 130 | Multivitamin + Minerals, 100 tabs | Pk |  |  |  |  |  |  |  |  |  |
| 131 | Oral rehydration salts | Sachet |  |  |  |  |  |  |  |  |  |
| 132 | Paracetamol 100mg, 10 supp | Pk |  |  |  |  |  |  |  |  |  |
| 133 | Paracetamol 120mg/5ml, 100ml syrup | Bottle |  |  |  |  |  |  |  |  |  |
| 134 | Paracetamol 500mg, 1000 tabs | Pk |  |  |  |  |  |  |  |  |  |
| 135 | Penicillin G, Benzathine 2.4MIU inj. | Vial |  |  |  |  |  |  |  |  |  |
| 136 | Prednisolone 5mg, 1000 tabs | Pk |  |  |  |  |  |  |  |  |  |
| 137 | Primaquine 15mg, 1000 tabs | Pk |  |  |  |  |  |  |  |  |  |
| 138 | Primaquine 7.5mg, 1000 tabs | Pk |  |  |  |  |  |  |  |  |  |
| 139 | Procaine penicillin 4MIU inj. | Vial |  |  |  |  |  |  |  |  |  |
| 140 | Pyridoxine 50mg, 1000 tabs | Pk |  |  |  |  |  |  |  |  |  |
| 141 | Pyrimethamine 25mg, 100 tabs | Pk |  |  |  |  |  |  |  |  |  |
| 142 | Sodium chloride 0.9%, Normal saline IV infusion, 1000 ml | Bag |  |  |  |  |  |  |  |  |  |
| 143 | Sulfadizine 500mg, 100 tabs | Pk |  |  |  |  |  |  |  |  |  |
| 144 | Sulphamethoxazole + Trimetroprim 200mg+40mg/5ml (Cotrimoxazole), 100ml susp | Bottle |  |  |  |  |  |  |  |  |  |
| 145 | Sulphamethoxazole 400mg + Trimetroprim 80mg (Cotrimoxazole), 1000 tabs | Pk |  |  |  |  |  |  |  |  |  |
| 146 | Sulphamethoxazole 800mg+Trimetroprim 160mg (Cotrimoxazole), 1000 tabs | Bottle |  |  |  |  |  |  |  |  |  |
| 147 | Thiabendazole 100mg/ml, 30ml susp | Bottle |  |  |  |  |  |  |  |  |  |
| 148 | Thiabendazole 500mg, 1000 tabs | Pk |  |  |  |  |  |  |  |  |  |
| 149 | Water for injection 10ml | Vial |  |  |  |  |  |  |  |  |  |
| 150 | Water for injection 5ml | Vial |  |  |  |  |  |  |  |  |  |
| 151 |  |  |  |  |  |  |  |  |  |  |  |
| 152 |  |  |  |  |  |  |  |  |  |  |  |
| 153 |  |  |  |  |  |  |  |  |  |  |  |
| 154 |  |  |  |  |  |  |  |  |  |  |  |
| 155 |  |  |  |  |  |  |  |  |  |  |  |
| 156 |  |  |  |  |  |  |  |  |  |  |  |
| 157 |  |  |  |  |  |  |  |  |  |  |  |
| 158 |  |  |  |  |  |  |  |  |  |  |  |
| 159 |  |  |  |  |  |  |  |  |  |  |  |
| 160 |  |  |  |  |  |  |  |  |  |  |  |
| 161 |  |  |  |  |  |  |  |  |  |  |  |
| 162 |  |  |  |  |  |  |  |  |  |  |  |
| 163 |  |  |  |  |  |  |  |  |  |  |  |
| 164 |  |  |  |  |  |  |  |  |  |  |  |
| 165 |  |  |  |  |  |  |  |  |  |  |  |
| 166 |  |  |  |  |  |  |  |  |  |  |  |
| 167 |  |  |  |  |  |  |  |  |  |  |  |
| 168 |  |  |  |  |  |  |  |  |  |  |  |
| 169 |  |  |  |  |  |  |  |  |  |  |  |
| 170 |  |  |  |  |  |  |  |  |  |  |  |
| 171 |  |  |  |  |  |  |  |  |  |  |  |
| 172 |  |  |  |  |  |  |  |  |  |  |  |

**TABLE 10.2 PROGRAM REAGENTS, SUPPLIES AND DIAGNOSTICS (FOR NON-LAB MONITORING HEALTH CENTERS)**

| **Period** | | | **1** | **2** | **3** | **4** | **5** | **6** | **7** | **8** | **9** |
| --- | --- | --- | --- | --- | --- | --- | --- | --- | --- | --- | --- |
| **BEGINNING DATE🡪** | | |  |  |  |  |  |  |  |  |  |
| **ENDING DATE🡪** | | |  |  |  |  |  |  |  |  |  |
| **Ser. No.** | **Product description** | **Unit of issue** |  |  |  |  |  |  |  |  |  |
|  | 1. **EID Supplies (HPCP I)** |  |  |  |  |  |  |  |  |  |  |
| 1 | Dry Blood Spot (DBS) Sample Collection Bundle, 20 tests |  |  |  |  |  |  |  |  |  |  |
| 2 | Dry Blood Spot (DBS) Sample Collection Bundle, 50 tests |  |  |  |  |  |  |  |  |  |  |
| 3 | Powder free Glove |  |  |  |  |  |  |  |  |  |  |
|  | **HIV Rapid Tests (HPCP II)** |  |  |  |  |  |  |  |  |  |  |
| 4 | Capillary EDTA Tube, 50 ul, 100 pcs |  |  |  |  |  |  |  |  |  |  |
| 5 | Contact lancet, 1.5 X 2 mm 200 pcs |  |  |  |  |  |  |  |  |  |  |
| 6 | Contact lancet, 21G X 1.8 mm 200 pcs |  |  |  |  |  |  |  |  |  |  |
| 7 | KHB, 50 tests |  |  |  |  |  |  |  |  |  |  |
| 8 | Stat-Pack, 20 tests |  |  |  |  |  |  |  |  |  |  |
| 9 | Uni-gold, 20 tests |  |  |  |  |  |  |  |  |  |  |
|  | **OI Diagnostics Reagents (HPCP II)** |  |  |  |  |  |  |  |  |  |  |
| 10 | Acetone alcohol solution |  |  |  |  |  |  |  |  |  |  |
| 11 | Blood group reagents, 5 ml (Anti – A) |  |  |  |  |  |  |  |  |  |  |
| 12 | Blood group reagents, 5 ml (Anti – B) |  |  |  |  |  |  |  |  |  |  |
| 13 | Blood group reagents, 5 ml (Anti – D) |  |  |  |  |  |  |  |  |  |  |
| 14 | Cotton tip applicator, 200 pcs |  |  |  |  |  |  |  |  |  |  |
| 15 | Cryptococcal latex Ag test, 70 tests |  |  |  |  |  |  |  |  |  |  |
| 16 | Crystal Violet 1% solution |  |  |  |  |  |  |  |  |  |  |
| 17 | Filter paper, 100 pcs |  |  |  |  |  |  |  |  |  |  |
| 18 | Gram’s Iodine solution |  |  |  |  |  |  |  |  |  |  |
| 19 | HCl 0.2N (Hemoglobin) |  |  |  |  |  |  |  |  |  |  |
| 20 | HCl 1% (WBC diluting fluid) |  |  |  |  |  |  |  |  |  |  |
| 21 | Hepatitis B Surface antigen, 25 tests |  |  |  |  |  |  |  |  |  |  |
| 22 | Hepatitis C Surface antigen, 25 tests |  |  |  |  |  |  |  |  |  |  |
| 23 | Lens paper, 100 pcs |  |  |  |  |  |  |  |  |  |  |
| 24 | Methylene blue |  |  |  |  |  |  |  |  |  |  |
| 25 | Microscope Coverslips, 100 pcs |  |  |  |  |  |  |  |  |  |  |
| 26 | Microscope slide, non-frosted, 50 pcs |  |  |  |  |  |  |  |  |  |  |
| 27 | Oil immersion, 100ml |  |  |  |  |  |  |  |  |  |  |
| 28 | Potassium Hydroxide, 10% solution (KOH) |  |  |  |  |  |  |  |  |  |  |
| 29 | Pregnancy test, 100 tests |  |  |  |  |  |  |  |  |  |  |
| 30 | RPR Syphilis test, 100 tests |  |  |  |  |  |  |  |  |  |  |
| 31 | Safranine solution |  |  |  |  |  |  |  |  |  |  |
| 32 | Sodium citrate (3.8%) |  |  |  |  |  |  |  |  |  |  |
| 33 | Urinalysis test strip 100 pcs, 10 parameter |  |  |  |  |  |  |  |  |  |  |
|  | **TB Diagnostics Reagents (TBLPCP)** |  |  |  |  |  |  |  |  |  |  |
| 33 | Acid alcohol 3% solution | Liter |  |  |  |  |  |  |  |  |  |
| 34 | Carbol fuchsin solution | Liter |  |  |  |  |  |  |  |  |  |
| 35 | Filter paper, 100 pcs | Pk |  |  |  |  |  |  |  |  |  |
| 36 | Lens paper, 100 pcs | Pk |  |  |  |  |  |  |  |  |  |
| 37 | Methylene blue | Liter |  |  |  |  |  |  |  |  |  |
| 38 | Microscope Coverslips, 100 pcs | Pk |  |  |  |  |  |  |  |  |  |
| 39 | Microscope slide, non-frosted, 50 pcs | Pk |  |  |  |  |  |  |  |  |  |
| 40 | Oil immersion, 100ml | Bottle |  |  |  |  |  |  |  |  |  |
| 41 | Sputum Cup, 1000 pcs | Pk |  |  |  |  |  |  |  |  |  |
| 42 | Wooden applicator, 1000 pcs | Pk |  |  |  |  |  |  |  |  |  |
|  | **Malaria Diagnostics Reagents (MPCP)** |  |  |  |  |  |  |  |  |  |  |
| 40 | Contact lancet, 1.5 X 2 mm 200 pcs |  |  |  |  |  |  |  |  |  |  |
| 41 | Contact lancet, 21G X 1.8 mm 200 pcs |  |  |  |  |  |  |  |  |  |  |
| 42 | Filter paper, 100 pcs |  |  |  |  |  |  |  |  |  |  |
| 43 | Giemsa stain solution |  |  |  |  |  |  |  |  |  |  |
| 44 | Lens paper, 100 pcs |  |  |  |  |  |  |  |  |  |  |
| 45 | Microscope slide, non-frosted, 50 pcs |  |  |  |  |  |  |  |  |  |  |
| 46 | Oil immersion, 100ml |  |  |  |  |  |  |  |  |  |  |
| 47 | Rapid Diagnostic Test for Malaria (RDT), 20 tests |  |  |  |  |  |  |  |  |  |  |
|  | **Supplies and Consumables** |  |  |  |  |  |  |  |  |  |  |
|  | **F.1. IPs (HPCP I) I** |  |  |  |  |  |  |  |  |  |  |
| 48 | Adhesive plasters, 12.5cmX10m | Roll |  |  |  |  |  |  |  |  |  |
| 49 | Adhesive plasters, perforated, 5cmX5cm | Roll |  |  |  |  |  |  |  |  |  |
| 50 | Alcohol denatured, 70% solution | Liter |  |  |  |  |  |  |  |  |  |
| 51 | Apron, cotton reusable and autoclavable, different sizes | Each |  |  |  |  |  |  |  |  |  |
| 52 | Apron, synthetic different sizes | Each |  |  |  |  |  |  |  |  |  |
| 53 | Biohazard bag, 22cmX28cm, 50 pcs | Pk |  |  |  |  |  |  |  |  |  |
| 54 | Biohazard bag, 61cmX76cm, 100 pcs | Pk |  |  |  |  |  |  |  |  |  |
| 55 | Boots, different sizes | Pair |  |  |  |  |  |  |  |  |  |
| 56 | Chlorohexidine 1.5% + Cetrimide 15% (Savlon), 250ml | Bottle |  |  |  |  |  |  |  |  |  |
| 57 | Color coded waste segregation bin – (Black) – 360-400 lit. | Each |  |  |  |  |  |  |  |  |  |
| 58 | Color coded waste segregation bin – (Black) – 60 lit. | Each |  |  |  |  |  |  |  |  |  |
| 59 | Color coded waste segregation bin – (Yellow) – 360-400 lit. | Each |  |  |  |  |  |  |  |  |  |
| 60 | Color coded waste segregation bin – (Black) – 60 lit. | Each |  |  |  |  |  |  |  |  |  |
| 61 | Cotton absorbent, 100gm | Roll |  |  |  |  |  |  |  |  |  |
| 62 | Cotton tip applicator, 200 pcs | Pk |  |  |  |  |  |  |  |  |  |
| 63 | Cotton Wool, absorbant, 500 gm | Roll |  |  |  |  |  |  |  |  |  |
| 64 | Dust mask/protective respirator | Each |  |  |  |  |  |  |  |  |  |
| 65 | Face mask of 50 pcs | Pk |  |  |  |  |  |  |  |  |  |
| 66 | Face mask, with ear loops and sterilizable medium size, 50 pcs | Pk |  |  |  |  |  |  |  |  |  |
| 67 | Face shield, two-side anti fog coating | Each |  |  |  |  |  |  |  |  |  |
| 68 | Gauze 4cm x 4cm of 10 | Pk |  |  |  |  |  |  |  |  |  |
| 69 | Gauze bandage rolls, 12 x 5 cm, 12 roll | Pk |  |  |  |  |  |  |  |  |  |
| 70 | Gauze bandage, 10cmX3m | Roll |  |  |  |  |  |  |  |  |  |
| 71 | Gauze bandage, 7.5cmX5m | Roll |  |  |  |  |  |  |  |  |  |
| 72 | Gauze bandage, nonsterile, 90cmX90m | Roll |  |  |  |  |  |  |  |  |  |
| 73 | Gauze swab/compress gauze sterile | Pk |  |  |  |  |  |  |  |  |  |
| 74 | Glove, examination, latex and powdered, large, 100 pcs | Pk |  |  |  |  |  |  |  |  |  |
| 75 | Glove, examination, latex and powdered, medium, 100 pcs | Pk |  |  |  |  |  |  |  |  |  |
| 76 | Glove, examination, latex and powdered, small, 100 pcs | Pk |  |  |  |  |  |  |  |  |  |
| 77 | Gloves, gynaecological, elbow length, medium | Pair |  |  |  |  |  |  |  |  |  |
| 78 | Gloves, heavy duty, large | Pair |  |  |  |  |  |  |  |  |  |
| 79 | Gloves, heavy duty, medium | Pair |  |  |  |  |  |  |  |  |  |
| 80 | Gloves, heavy duty, small | Pair |  |  |  |  |  |  |  |  |  |
| 81 | Gloves, surgical, small (6.5-7), 50 pairs | Pk |  |  |  |  |  |  |  |  |  |
| 82 | Gloves, surgical, large (8), 50 pairs | Pk |  |  |  |  |  |  |  |  |  |
| 83 | Gloves, surgical, medium (7.5), 50 pairs | Pk |  |  |  |  |  |  |  |  |  |
| 84 |  |  |  |  |  |  |  |  |  |  |  |
| 85 |  |  |  |  |  |  |  |  |  |  |  |
| 86 |  |  |  |  |  |  |  |  |  |  |  |
| 87 |  |  |  |  |  |  |  |  |  |  |  |
| 88 |  |  |  |  |  |  |  |  |  |  |  |
| 89 |  |  |  |  |  |  |  |  |  |  |  |
| 90 |  |  |  |  |  |  |  |  |  |  |  |
| 91 |  |  |  |  |  |  |  |  |  |  |  |
|  | **F.1. IPs (HPCP I) II** |  |  |  |  |  |  |  |  |  |  |
| 92 | Gluteraldehide solution | Liter |  |  |  |  |  |  |  |  |  |
| 93 | Glycerin pure 98%^, 1 liter (Immulante) | Liter |  |  |  |  |  |  |  |  |  |
| 94 | Hair cover (capes) | Each |  |  |  |  |  |  |  |  |  |
| 95 | Hand wash liquid sap, 100 ml | ml |  |  |  |  |  |  |  |  |  |
| 96 | Hydrogen peroxide 3% solution, 1 liter (HLD) | Liter |  |  |  |  |  |  |  |  |  |
| 97 | Iodine preparation(3%), water or alcohol based, 1 liter (A) | Liter |  |  |  |  |  |  |  |  |  |
| 98 | Plastic buckets 20 lit. – for decontaminating instruments | Each |  |  |  |  |  |  |  |  |  |
| 99 | Povidone iodine 10% solution | Liter |  |  |  |  |  |  |  |  |  |
| 100 | Safety boxes, disposable, 5 lit. 25 pcs | Pk |  |  |  |  |  |  |  |  |  |
| 101 | Soap bar 200-250gm | Each |  |  |  |  |  |  |  |  |  |
| 102 | Sodium hypochlorite 5% solution | Liter |  |  |  |  |  |  |  |  |  |
| 103 | Syringe, Auto Disable with needle 10ml, 100 pcs | Pk |  |  |  |  |  |  |  |  |  |
| 104 | Syringe, Auto Disable with needle 2ml, 100 pcs | Pk |  |  |  |  |  |  |  |  |  |
| 105 | Syringe, Auto Disable with needle 3ml, 100 pcs | Pk |  |  |  |  |  |  |  |  |  |
| 106 | Syringe, Auto Disable with needle 5ml, 100 pcs | Pk |  |  |  |  |  |  |  |  |  |
| 107 | Syringe, disposable with needle 10ml, 100 pcs | Pk |  |  |  |  |  |  |  |  |  |
| 108 | Syringe, disposable with needle 2ml, 100 pcs | Pk |  |  |  |  |  |  |  |  |  |
| 109 | Syringe, disposable with needle 3ml, 100 pcs | Pk |  |  |  |  |  |  |  |  |  |
| 110 | Syringe, disposable with needle 5ml, 100 pcs | Pk |  |  |  |  |  |  |  |  |  |
| 111 | Vacutainer Holder of 1000 | Pk |  |  |  |  |  |  |  |  |  |
| 112 | Vacutainer Needle – 21G x 1.5’’, 1000/Pack | Pk |  |  |  |  |  |  |  |  |  |
| 113 | Vacutainer SSt tube of 1000/Pack | Pk |  |  |  |  |  |  |  |  |  |
| 114 | Vacutainer tube with EDTA of 1000– 4ml | Pk |  |  |  |  |  |  |  |  |  |
| 115 |  |  |  |  |  |  |  |  |  |  |  |
| 116 |  |  |  |  |  |  |  |  |  |  |  |
| 117 |  |  |  |  |  |  |  |  |  |  |  |
| 118 |  |  |  |  |  |  |  |  |  |  |  |
| 119 |  |  |  |  |  |  |  |  |  |  |  |
| 120 |  |  |  |  |  |  |  |  |  |  |  |
| 121 |  |  |  |  |  |  |  |  |  |  |  |
| 122 |  |  |  |  |  |  |  |  |  |  |  |
| 123 |  |  |  |  |  |  |  |  |  |  |  |
|  |  |  |  |  |  |  |  |  |  |  |  |
| 124 |  |  |  |  |  |  |  |  |  |  |  |
| 125 |  |  |  |  |  |  |  |  |  |  |  |
| 126 |  |  |  |  |  |  |  |  |  |  |  |
| 127 |  |  |  |  |  |  |  |  |  |  |  |
| 128 |  |  |  |  |  |  |  |  |  |  |  |
| 129 |  |  |  |  |  |  |  |  |  |  |  |
| 130 |  |  |  |  |  |  |  |  |  |  |  |
| 131 |  |  |  |  |  |  |  |  |  |  |  |
| 132 |  |  |  |  |  |  |  |  |  |  |  |
|  | **2. Lab Consumables (HPCP II)** |  |  |  |  |  |  |  |  |  |  |
| 133 | Bag Biohazard Autoclavable of 100pcs | Pk |  |  |  |  |  |  |  |  |  |
| 134 | Butterfly needle 23’’ or Safety lock blood collection set, 200 pcs | Pk |  |  |  |  |  |  |  |  |  |
| 135 | Cryogenic 1.8 ml, 450 vials | Pk |  |  |  |  |  |  |  |  |  |
| 136 | Face masks, with earl loops, 50 pcs | Pk |  |  |  |  |  |  |  |  |  |
| 137 | Flash back needle 21G x 1’’, 50 pcs | Pk |  |  |  |  |  |  |  |  |  |
| 138 | Glove Nitrile of 50pairs Large | Pk |  |  |  |  |  |  |  |  |  |
| 139 | Glove Nitrile of 50pairs Medium | Pk |  |  |  |  |  |  |  |  |  |
| 140 | Glove Nitrile of 50pairs Small | Pk |  |  |  |  |  |  |  |  |  |
| 141 | Gloves, Latex, Powdered, Large, 100 Pcs | Pk |  |  |  |  |  |  |  |  |  |
| 142 | Gloves, Latex, Powdered, Medium, 100 Pcs | Pk |  |  |  |  |  |  |  |  |  |
| 143 | Gloves, Latex, Powdered, Small, 100 Pcs | Pk |  |  |  |  |  |  |  |  |  |
| 144 | Microtainer tube K2 EDTA 250-400ul, 100 pcs | Pk |  |  |  |  |  |  |  |  |  |
| 145 | Microtainer tube SST, 200 pcs | Pk |  |  |  |  |  |  |  |  |  |
| 146 | Pasteur pipette, non-sterile, 3ml, 500 pcs | Pk |  |  |  |  |  |  |  |  |  |
| 147 | Pipette tips 1000ul, non-filtered blue, 1000pcs | Pk |  |  |  |  |  |  |  |  |  |
| 148 | Pipette tips 200ul, non-filtered yellow, 1000 pcs | Pk |  |  |  |  |  |  |  |  |  |
| 149 | Safety box, 25 pcs | Pk |  |  |  |  |  |  |  |  |  |
| 150 | Sodium hypochlorite solution, liter | Liter |  |  |  |  |  |  |  |  |  |
| 151 | Vacutainer multi sample needle 21 g, 1000 pcs | Pk |  |  |  |  |  |  |  |  |  |
| 152 | Vacutainer one use holders, 100 pcs | Pk |  |  |  |  |  |  |  |  |  |
| 153 | Vacutainer serum separator tube (SST) 5ml, 1000 pcs | Pk |  |  |  |  |  |  |  |  |  |
| 154 | Vacutainer tubes PLK2 E, EDTA or purple top 5ml, 1000pcs | Pk |  |  |  |  |  |  |  |  |  |
| 155 | Vacutainer tubes PLK2 E, EDTRA or purple top 5ml, 1000 pcs | Pk |  |  |  |  |  |  |  |  |  |
| 156 | Vacutainer tubes PLK3E, EDTA or purple top 5ml, 1000 pcs | Pk |  |  |  |  |  |  |  |  |  |
| 157 |  |  |  |  |  |  |  |  |  |  |  |
| 158 |  |  |  |  |  |  |  |  |  |  |  |
| 159 |  |  |  |  |  |  |  |  |  |  |  |
| 160 |  |  |  |  |  |  |  |  |  |  |  |
| 161 |  |  |  |  |  |  |  |  |  |  |  |
| 162 |  |  |  |  |  |  |  |  |  |  |  |
| 163 |  |  |  |  |  |  |  |  |  |  |  |
| 164 |  |  |  |  |  |  |  |  |  |  |  |
| 165 |  |  |  |  |  |  |  |  |  |  |  |
| 166 |  |  |  |  |  |  |  |  |  |  |  |
| 167 |  |  |  |  |  |  |  |  |  |  |  |
| 168 |  |  |  |  |  |  |  |  |  |  |  |
| 169 |  |  |  |  |  |  |  |  |  |  |  |
| 170 |  |  |  |  |  |  |  |  |  |  |  |
| 171 |  |  |  |  |  |  |  |  |  |  |  |
| 172 |  |  |  |  |  |  |  |  |  |  |  |
| 173 |  |  |  |  |  |  |  |  |  |  |  |
| 174 |  |  |  |  |  |  |  |  |  |  |  |
| 175 |  |  |  |  |  |  |  |  |  |  |  |
| 176 |  |  |  |  |  |  |  |  |  |  |  |
| 177 |  |  |  |  |  |  |  |  |  |  |  |
| 178 |  |  |  |  |  |  |  |  |  |  |  |
| 179 |  |  |  |  |  |  |  |  |  |  |  |
|  |  |  |  |  |  |  |  |  |  |  |  |
|  |  |  |  |  |  |  |  |  |  |  |  |

**TABLE 10.3 PROGRAM REAGENTS, SUPPLIES AND DIAGNOSTICS (FOR LAB MONITORING HEALTH CENTERS)**

| **Period** | | | **1** | **2** | **3** | **4** | **5** | **6** | **7** | **8** | **9** |
| --- | --- | --- | --- | --- | --- | --- | --- | --- | --- | --- | --- |
| **BEGINNING DATE🡪** | | |  |  |  |  |  |  |  |  |  |
| **ENDING DATE🡪** | | |  |  |  |  |  |  |  |  |  |
| **Ser. No.** | **Product description** | **Unit of issue** |  |  |  |  |  |  |  |  |  |
|  | 1. **ART Lab reagents (HPCP I)** |  |  |  |  |  |  |  |  |  |  |
|  | **A.1. CD4 Reagents** |  |  |  |  |  |  |  |  |  |  |
| 1 | BD FACS Count – CD3/4 Reagent Kit (Single tube), 50 tests |  |  |  |  |  |  |  |  |  |  |
| 2 | BD FACS Count – CD3/8/4 Reagent Kit (Double tube), 50 tests |  |  |  |  |  |  |  |  |  |  |
| 3 | BD FACS Count – Control Kit, 25 tests |  |  |  |  |  |  |  |  |  |  |
| 4 | BD FACS Count – Facs Rinse, 5 Lit. |  |  |  |  |  |  |  |  |  |  |
| 5 | BD FACS Count – Facs Clean, 5 Lit. |  |  |  |  |  |  |  |  |  |  |
| 6 | BD FACS Count – Facs Flow, 20 Lit. |  |  |  |  |  |  |  |  |  |  |
| 7 | BD FACS Count – Thermal Paper roll |  |  |  |  |  |  |  |  |  |  |
| 8 | Cleaning tube of 100 pcs |  |  |  |  |  |  |  |  |  |  |
| 9 | Falcon tube, 5ml of 100pcs |  |  |  |  |  |  |  |  |  |  |
| 10 |  |  |  |  |  |  |  |  |  |  |  |
| 11 |  |  |  |  |  |  |  |  |  |  |  |
| 12 |  |  |  |  |  |  |  |  |  |  |  |
|  | **A.2. Chemistry reagents** |  |  |  |  |  |  |  |  |  |  |
| 13 | Alkaline Phosphatase 8x50ml, 400ml |  |  |  |  |  |  |  |  |  |  |
| 14 | Alpha amylase, 12x10ml, 120ml |  |  |  |  |  |  |  |  |  |  |
| 15 | Auto creatinine, 250ml |  |  |  |  |  |  |  |  |  |  |
| 16 | Autocal or Clinical Chemistry multi-calibrator, 4x5ml, 20ml |  |  |  |  |  |  |  |  |  |  |
| 17 | Bilirubin Direct, 375ml |  |  |  |  |  |  |  |  |  |  |
| 18 | Bilirubin Total, 375ml |  |  |  |  |  |  |  |  |  |  |
| 19 | Cholesterol, 4x100ml, 400ml |  |  |  |  |  |  |  |  |  |  |
| 20 | Control nomrla.Humatrol N, 6x5ml, 30ml |  |  |  |  |  |  |  |  |  |  |
| 21 | Control pathological/Humatrol P, 6x5ml, 30ml |  |  |  |  |  |  |  |  |  |  |
| 22 | Glucose 4x100ml, 400ml |  |  |  |  |  |  |  |  |  |  |
| 23 | GOT/AST, 8x50ml, 400ml |  |  |  |  |  |  |  |  |  |  |
| 24 | GPT/ALT, 8x50ml, 400ml |  |  |  |  |  |  |  |  |  |  |
| 25 | Triglycerides, 4x100ml, 400ml |  |  |  |  |  |  |  |  |  |  |
| 26 | Urea, 8x50ml, 400ml |  |  |  |  |  |  |  |  |  |  |
| 27 |  |  |  |  |  |  |  |  |  |  |  |
| 28 |  |  |  |  |  |  |  |  |  |  |  |
|  | **A.3. Hematology** |  |  |  |  |  |  |  |  |  |  |
| 29 | Cell-Dyn 1800 – CN-free HGB/WIC (diff) lyse, 3.8liters |  |  |  |  |  |  |  |  |  |  |
| 30 | Cell-Dyn 1800 – Detergent solution, 20 lit. |  |  |  |  |  |  |  |  |  |  |
| 31 | Cell-Dyn 1800 – Isotonic diluent, 20 lit. |  |  |  |  |  |  |  |  |  |  |
| 32 | Cell-Dyn Enzymatic cleaner, 2x50ml, 100ml |  |  |  |  |  |  |  |  |  |  |
| 33 | Sysmex – Cell clean, 50 ml |  |  |  |  |  |  |  |  |  |  |
| 34 | Sysmex – Cell pack, 20 lit. |  |  |  |  |  |  |  |  |  |  |
| 35 | Sysmex KX21 – Thermal Printer paper |  |  |  |  |  |  |  |  |  |  |
| 36 | Sysmex KX-21N – Stromatolyser SWH- 200A, 1500ml |  |  |  |  |  |  |  |  |  |  |
| 37 |  |  |  |  |  |  |  |  |  |  |  |
| 38 |  |  |  |  |  |  |  |  |  |  |  |
|  | **A.4. EID Supplies** |  |  |  |  |  |  |  |  |  |  |
| 39 | Dry Blood Spot (DBS) Sample Collection Bundle, 20 tests |  |  |  |  |  |  |  |  |  |  |
| 40 | Dry Blood Spot (DBS) Sample Collection Bundle, 50 tests |  |  |  |  |  |  |  |  |  |  |
| 41 |  |  |  |  |  |  |  |  |  |  |  |
| 42 |  |  |  |  |  |  |  |  |  |  |  |
| 43 |  |  |  |  |  |  |  |  |  |  |  |
|  | **B. HIV Rapid Tests (HPCP II)** |  |  |  |  |  |  |  |  |  |  |
| 44 | Capillary EDTA Tube, 50ul, 100 pcs |  |  |  |  |  |  |  |  |  |  |
| 45 | Contact lancet, 1.5 x 2 mm 200 pcs |  |  |  |  |  |  |  |  |  |  |
| 46 | Contact lancet, 21G x 1.8mm 200 pcs |  |  |  |  |  |  |  |  |  |  |
| 47 | KHB, 50 tests |  |  |  |  |  |  |  |  |  |  |
| 48 | Stat-Pack, 20 tests |  |  |  |  |  |  |  |  |  |  |
| 49 | Uni-gold, 20 tests |  |  |  |  |  |  |  |  |  |  |
| 50 |  |  |  |  |  |  |  |  |  |  |  |
| 51 |  |  |  |  |  |  |  |  |  |  |  |
|  | **C. OI Diagnostics Reagents (HPCP III)** |  |  |  |  |  |  |  |  |  |  |
| 52 | Acetone alcohol solution |  |  |  |  |  |  |  |  |  |  |
| 53 | Blood group reagents, 5 ml (Anti – A) |  |  |  |  |  |  |  |  |  |  |
| 54 | Blood group reagents, 5 ml (Anti – B) |  |  |  |  |  |  |  |  |  |  |
| 55 | Blood group reagents, 5 ml (Anti – D) |  |  |  |  |  |  |  |  |  |  |
| 56 | Cotton tip applicator, 200 pcs |  |  |  |  |  |  |  |  |  |  |
| 57 | Cryptococcal latex Ag test, 70 tests |  |  |  |  |  |  |  |  |  |  |
| 58 | Crystal violet 1% solution |  |  |  |  |  |  |  |  |  |  |
| 59 | Gram’s Iodine solution |  |  |  |  |  |  |  |  |  |  |
| 60 | HCl 0.1N (Hemoglobin) |  |  |  |  |  |  |  |  |  |  |
| 61 | HCl 1% (WBC diluting fluid) |  |  |  |  |  |  |  |  |  |  |
| 62 | Hepatitis B Surface antigen, 25 tests |  |  |  |  |  |  |  |  |  |  |
| 63 | Hepatitis C Surface antigen, 25 tests |  |  |  |  |  |  |  |  |  |  |
| 64 | Lens paper, 100 pcs |  |  |  |  |  |  |  |  |  |  |
| 65 | Methylene blue |  |  |  |  |  |  |  |  |  |  |
| 66 | Microscope Coverslips, 100 pcs |  |  |  |  |  |  |  |  |  |  |
| 67 | Microscope slide, non-frosted, 50 pcs |  |  |  |  |  |  |  |  |  |  |
| 68 | Oil immersion, 100ml |  |  |  |  |  |  |  |  |  |  |
| 69 | Potassium Hydroxide, 10% solution (KOH) |  |  |  |  |  |  |  |  |  |  |
| 70 | Pregnancy test, 100 tests |  |  |  |  |  |  |  |  |  |  |
| 71 | RPR Syphilis test, 100 tests |  |  |  |  |  |  |  |  |  |  |
| 72 | Safranine solution |  |  |  |  |  |  |  |  |  |  |
| 73 | Urinalysis test strip 100 pcs, 10 parameter |  |  |  |  |  |  |  |  |  |  |
| 74 |  |  |  |  |  |  |  |  |  |  |  |
|  | **D. TB Diagnostics Reagents (TBLPCP)** |  |  |  |  |  |  |  |  |  |  |
| 75 | Acid alcohol 3% solution | Liter |  |  |  |  |  |  |  |  |  |
| 76 | Carbol fuchsin solution | Liter |  |  |  |  |  |  |  |  |  |
| 77 | Filter paper, 100 pcs | Pk |  |  |  |  |  |  |  |  |  |
| 78 | Lens paper, 100 pcs | Pk |  |  |  |  |  |  |  |  |  |
| 79 | Methylene blue | Liter |  |  |  |  |  |  |  |  |  |
| 80 | Microscope Coverslips, 100 pcs | Pk |  |  |  |  |  |  |  |  |  |
| 81 | Microscope slide, non-frosted, 50 pcs | Pk |  |  |  |  |  |  |  |  |  |
| 82 | Oil immersion, 100ml | Bottle |  |  |  |  |  |  |  |  |  |
| 83 | Sputum Cup, 1000 pcs | Pk |  |  |  |  |  |  |  |  |  |
| 84 | Wooden applicator, 1000 pcs | Pk |  |  |  |  |  |  |  |  |  |
| 85 |  |  |  |  |  |  |  |  |  |  |  |
| 86 |  |  |  |  |  |  |  |  |  |  |  |
|  | **Supplies and Consumables** |  |  |  |  |  |  |  |  |  |  |
|  | **F.1. IPs (HPCP I) I** |  |  |  |  |  |  |  |  |  |  |
| 87 | Adhesive plasters, 12.5cmX10m | Roll |  |  |  |  |  |  |  |  |  |
| 88 | Adhesive plasters, perforated, 5cmX5cm | Roll |  |  |  |  |  |  |  |  |  |
| 89 | Alcohol denatured, 70% solution | Liter |  |  |  |  |  |  |  |  |  |
| 90 | Apron, cotton reusable and autoclavable, different sizes | Each |  |  |  |  |  |  |  |  |  |
| 91 | Apron, synthetic different sizes | Each |  |  |  |  |  |  |  |  |  |
| 92 | Biohazard bag, 22cmX28cm, 50 pcs | Pk |  |  |  |  |  |  |  |  |  |
| 93 | Biohazard bag, 61cmX76cm, 100 pcs | Pk |  |  |  |  |  |  |  |  |  |
| 94 | Boots, different sizes | Pair |  |  |  |  |  |  |  |  |  |
| 95 | Chlorohexidine 1.5% + Cetrimide 15% (Savlon), 250ml | Bottle |  |  |  |  |  |  |  |  |  |
| 96 | Color coded waste segregation bin – (Black) – 360-400 lit. | Each |  |  |  |  |  |  |  |  |  |
| 97 | Color coded waste segregation bin – (Black) – 60 lit. | Each |  |  |  |  |  |  |  |  |  |
| 98 | Color coded waste segregation bin – (Yellow) – 360-400 lit. | Each |  |  |  |  |  |  |  |  |  |
| 99 | Color coded waste segregation bin – (Black) – 60 lit. | Each |  |  |  |  |  |  |  |  |  |
| 100 | Cotton absorbent, 100gm | Roll |  |  |  |  |  |  |  |  |  |
| 101 | Cotton tip applicator, 200 pcs | Pk |  |  |  |  |  |  |  |  |  |
| 102 | Cotton Wool, absorbant, 500 gm | Roll |  |  |  |  |  |  |  |  |  |
| 103 | Dust mask/protective respirator | Each |  |  |  |  |  |  |  |  |  |
| 104 | Face mask of 50 pcs | Pk |  |  |  |  |  |  |  |  |  |
| 105 | Face mask, with ear loops and sterilizable medium size, 50 pcs | Pk |  |  |  |  |  |  |  |  |  |
| 106 | Face shield, two-side anti fog coating | Each |  |  |  |  |  |  |  |  |  |
| 107 | Gauze 4cm x 4cm of 10 | Pk |  |  |  |  |  |  |  |  |  |
| 108 | Gauze bandage rolls, 12 x 5 cm, 12 roll | Pk |  |  |  |  |  |  |  |  |  |
| 109 | Gauze bandage, 10cmX3m | Roll |  |  |  |  |  |  |  |  |  |
| 110 | Gauze bandage, 7.5cmX5m | Roll |  |  |  |  |  |  |  |  |  |
| 111 | Gauze bandage, nonsterile, 90cmX90m | Roll |  |  |  |  |  |  |  |  |  |
| 112 | Gauze swab/compress gauze sterile | Pk |  |  |  |  |  |  |  |  |  |
| 113 | Glove, examination, latex and powdered, large, 100 pcs | Pk |  |  |  |  |  |  |  |  |  |
| 114 | Glove, examination, latex and powdered, medium, 100 pcs | Pk |  |  |  |  |  |  |  |  |  |
| 115 | Glove, examination, latex and powdered, small, 100 pcs | Pk |  |  |  |  |  |  |  |  |  |
| 116 | Gloves, gynaecological, elbow length, medium | Pair |  |  |  |  |  |  |  |  |  |
| 117 | Gloves, heavy duty, large | Pair |  |  |  |  |  |  |  |  |  |
| 118 | Gloves, heavy duty, medium | Pair |  |  |  |  |  |  |  |  |  |
| 119 | Gloves, heavy duty, small | Pair |  |  |  |  |  |  |  |  |  |
| 120 | Gloves, surgical, small (6.5-7), 50 pairs | Pk |  |  |  |  |  |  |  |  |  |
| 121 | Gloves, surgical, large (8), 50 pairs | Pk |  |  |  |  |  |  |  |  |  |
| 122 | Gloves, surgical, medium (7.5), 50 pairs | Pk |  |  |  |  |  |  |  |  |  |
| 123 |  |  |  |  |  |  |  |  |  |  |  |
| 124 |  |  |  |  |  |  |  |  |  |  |  |
| 125 |  |  |  |  |  |  |  |  |  |  |  |
| 126 |  |  |  |  |  |  |  |  |  |  |  |
| 127 |  |  |  |  |  |  |  |  |  |  |  |
| 128 |  |  |  |  |  |  |  |  |  |  |  |
| 129 |  |  |  |  |  |  |  |  |  |  |  |
| 130 |  |  |  |  |  |  |  |  |  |  |  |
| 131 |  |  |  |  |  |  |  |  |  |  |  |
| 132 |  |  |  |  |  |  |  |  |  |  |  |
| 133 |  |  |  |  |  |  |  |  |  |  |  |
| 134 |  |  |  |  |  |  |  |  |  |  |  |
| 135 |  |  |  |  |  |  |  |  |  |  |  |
| 136 |  |  |  |  |  |  |  |  |  |  |  |
| 137 |  |  |  |  |  |  |  |  |  |  |  |
| 138 |  |  |  |  |  |  |  |  |  |  |  |
| 139 |  |  |  |  |  |  |  |  |  |  |  |
| 140 |  |  |  |  |  |  |  |  |  |  |  |
| 141 |  |  |  |  |  |  |  |  |  |  |  |
| 142 |  |  |  |  |  |  |  |  |  |  |  |
| 143 |  |  |  |  |  |  |  |  |  |  |  |
|  | **F.1. IPs (HPCP I) II** |  |  |  |  |  |  |  |  |  |  |
| 144 | Gluteraldehide solution | Liter |  |  |  |  |  |  |  |  |  |
| 145 | Glycerin pure 98%^, 1 liter (Immulante) | Liter |  |  |  |  |  |  |  |  |  |
| 146 | Hair cover (capes) | Each |  |  |  |  |  |  |  |  |  |
| 147 | Hand wash liquid sap, 100 ml | ml |  |  |  |  |  |  |  |  |  |
| 148 | Hydrogen peroxide 3% solution, 1 liter (HLD) | Liter |  |  |  |  |  |  |  |  |  |
| 149 | Iodine preparation(3%), water or alcohol based, 1 liter (A) | Liter |  |  |  |  |  |  |  |  |  |
| 150 | Plastic buckets 20 lit. – for decontaminating instruments | Each |  |  |  |  |  |  |  |  |  |
| 151 | Povidone iodine 10% solution | Liter |  |  |  |  |  |  |  |  |  |
| 152 | Safety boxes, disposable, 5 lit. 25 pcs | Pk |  |  |  |  |  |  |  |  |  |
| 153 | Soap bar 200-250gm | Each |  |  |  |  |  |  |  |  |  |
| 154 | Sodium hypochlorite 5% solution | Liter |  |  |  |  |  |  |  |  |  |
| 155 | Syringe, Auto Disable with needle 10ml, 100 pcs | Pk |  |  |  |  |  |  |  |  |  |
| 156 | Syringe, Auto Disable with needle 2ml, 100 pcs | Pk |  |  |  |  |  |  |  |  |  |
| 157 | Syringe, Auto Disable with needle 3ml, 100 pcs | Pk |  |  |  |  |  |  |  |  |  |
| 158 | Syringe, Auto Disable with needle 5ml, 100 pcs | Pk |  |  |  |  |  |  |  |  |  |
| 159 | Syringe, disposable with needle 10ml, 100 pcs | Pk |  |  |  |  |  |  |  |  |  |
| 160 | Syringe, disposable with needle 2ml, 100 pcs | Pk |  |  |  |  |  |  |  |  |  |
| 161 | Syringe, disposable with needle 3ml, 100 pcs | Pk |  |  |  |  |  |  |  |  |  |
| 162 | Syringe, disposable with needle 5ml, 100 pcs | Pk |  |  |  |  |  |  |  |  |  |
| 163 | Vacutainer Holder of 1000 | Pk |  |  |  |  |  |  |  |  |  |
| 164 | Vacutainer Needle – 21G x 1.5’’, 1000/Pack | Pk |  |  |  |  |  |  |  |  |  |
| 165 | Vacutainer SSt tube of 1000/Pack | Pk |  |  |  |  |  |  |  |  |  |
| 166 | Vacutainer tube with EDTA of 1000– 4ml | Pk |  |  |  |  |  |  |  |  |  |
| 167 |  |  |  |  |  |  |  |  |  |  |  |
| 168 |  |  |  |  |  |  |  |  |  |  |  |
| 169 |  |  |  |  |  |  |  |  |  |  |  |
| 170 |  |  |  |  |  |  |  |  |  |  |  |
| 171 |  |  |  |  |  |  |  |  |  |  |  |
| 172 |  |  |  |  |  |  |  |  |  |  |  |
| 173 |  |  |  |  |  |  |  |  |  |  |  |
| 174 |  |  |  |  |  |  |  |  |  |  |  |
| 175 |  |  |  |  |  |  |  |  |  |  |  |
| 176 |  |  |  |  |  |  |  |  |  |  |  |
| 177 |  |  |  |  |  |  |  |  |  |  |  |
| 178 |  |  |  |  |  |  |  |  |  |  |  |
| 179 |  |  |  |  |  |  |  |  |  |  |  |
| 180 |  |  |  |  |  |  |  |  |  |  |  |
| 181 |  |  |  |  |  |  |  |  |  |  |  |
| 182 |  |  |  |  |  |  |  |  |  |  |  |
| 183 |  |  |  |  |  |  |  |  |  |  |  |
| 184 |  |  |  |  |  |  |  |  |  |  |  |
| 185 |  |  |  |  |  |  |  |  |  |  |  |
| 186 |  |  |  |  |  |  |  |  |  |  |  |
| 187 |  |  |  |  |  |  |  |  |  |  |  |
| 188 |  |  |  |  |  |  |  |  |  |  |  |
| 189 |  |  |  |  |  |  |  |  |  |  |  |
| 190 |  |  |  |  |  |  |  |  |  |  |  |
|  | **2. Lab Consumables (HPCP II)** |  |  |  |  |  |  |  |  |  |  |
| 191 | Bag Biohazard Autoclavable of 100pcs | Pk |  |  |  |  |  |  |  |  |  |
| 192 | Butterfly needle 23’’ or Safety lock blood collection set, 200 pcs | Pk |  |  |  |  |  |  |  |  |  |
| 193 | Cryogenic 1.8 ml, 450 vials | Pk |  |  |  |  |  |  |  |  |  |
| 194 | Face masks, with earl loops, 50 pcs | Pk |  |  |  |  |  |  |  |  |  |
| 195 | Flash back needle 21G x 1’’, 50 pcs | Pk |  |  |  |  |  |  |  |  |  |
| 196 | Glove Nitrile of 50pairs Large | Pk |  |  |  |  |  |  |  |  |  |
| 197 | Glove Nitrile of 50pairs Medium | Pk |  |  |  |  |  |  |  |  |  |
| 198 | Glove Nitrile of 50pairs Small | Pk |  |  |  |  |  |  |  |  |  |
| 199 | Gloves, Latex, Powdered, Large, 100 Pcs | Pk |  |  |  |  |  |  |  |  |  |
| 200 | Gloves, Latex, Powdered, Medium, 100 Pcs | Pk |  |  |  |  |  |  |  |  |  |
| 201 | Gloves, Latex, Powdered, Small, 100 Pcs | Pk |  |  |  |  |  |  |  |  |  |
| 202 | Microtainer tube K2 EDTA 250-400ul, 100 pcs | Pk |  |  |  |  |  |  |  |  |  |
| 203 | Microtainer tube SST, 200 pcs | Pk |  |  |  |  |  |  |  |  |  |
| 204 | Pasteur pipette, non-sterile, 3ml, 500 pcs | Pk |  |  |  |  |  |  |  |  |  |
| 205 | Pipette tips 1000ul, non-filtered blue, 1000pcs | Pk |  |  |  |  |  |  |  |  |  |
| 206 | Pipette tips 200ul, non-filtered yellow, 1000 pcs | Pk |  |  |  |  |  |  |  |  |  |
| 207 | Safety box, 25 pcs | Pk |  |  |  |  |  |  |  |  |  |
| 208 | Sodium hypochlorite solution, liter | Liter |  |  |  |  |  |  |  |  |  |
| 209 | Vacutainer multi sample needle 21 g, 1000 pcs | Pk |  |  |  |  |  |  |  |  |  |
| 210 | Vacutainer one use holders, 100 pcs | Pk |  |  |  |  |  |  |  |  |  |
| 211 | Vacutainer serum separator tube (SST) 5ml, 1000 pcs | Pk |  |  |  |  |  |  |  |  |  |
| 212 | Vacutainer tubes PLK2 E, EDTA or purple top 5ml, 1000pcs | Pk |  |  |  |  |  |  |  |  |  |
| 213 | Vacutainer tubes PLK2 E, EDTRA or purple top 5ml, 1000 pcs | Pk |  |  |  |  |  |  |  |  |  |
| 214 | Vacutainer tubes PLK3E, EDTA or purple top 5ml, 1000 pcs | Pk |  |  |  |  |  |  |  |  |  |
|  | **G. Supplies and Reagents (FHP)** |  |  |  |  |  |  |  |  |  |  |
| 215 | Glove, Sterile Latex Sugical, Size 8, 100 pcs | Pk |  |  |  |  |  |  |  |  |  |
| 216 | Cotton Absorbent 100 gm | Pk |  |  |  |  |  |  |  |  |  |
| 217 | Iodine Tincture 2% Solution of 1000 ml | Bottle |  |  |  |  |  |  |  |  |  |
| 218 | Syringe with needle 5ml, 100 pcs | Box |  |  |  |  |  |  |  |  |  |
| 219 |  |  |  |  |  |  |  |  |  |  |  |
| 220 |  |  |  |  |  |  |  |  |  |  |  |
| 221 |  |  |  |  |  |  |  |  |  |  |  |
| 222 |  |  |  |  |  |  |  |  |  |  |  |
| 223 |  |  |  |  |  |  |  |  |  |  |  |
| 224 |  |  |  |  |  |  |  |  |  |  |  |
| 225 |  |  |  |  |  |  |  |  |  |  |  |
| 226 |  |  |  |  |  |  |  |  |  |  |  |
| 227 |  |  |  |  |  |  |  |  |  |  |  |
| 228 |  |  |  |  |  |  |  |  |  |  |  |
| 229 |  |  |  |  |  |  |  |  |  |  |  |
| 230 |  |  |  |  |  |  |  |  |  |  |  |

**Section 2: Quantity of drugs distributed to health posts by the health center during the F.Y. 2006**

Instructions:

1. This section has to be completed with the person in-charge of the drug store or his/her representative.
2. Ask verbatim, Question 10.4 from the person in-charge
3. If the answer to 10.4 is “No”, skip to **next section**
4. List of steps to be followed if the answer to 10.4 is “Yes”:
   1. List down the health posts to which the health center distributed drugs and supplies during F.Y. 2006, on a blank piece of paper
   2. Ask the person in-charge “*Which one of these health posts is the most typical, for the supplies that this health center gives to the health posts?”* Record the name in 10.5a

**OR**

- 1. If the person in-charge is not able to respond to the above question, randomly select one health post from this list. Record the name in 10.5a
  2. Ask the person in-charge for **Model 22** for the entire F.Y. 2006 (Hamle 2005 – Sene 2006). There can be multiple pads / books of Model 22 for the whole year. Model 22 is the standard recording format centrally prepared and enforced by the Ministry of Finance and Economic Development (MOFED), to be used by all the drug stores for any drug disbursements.
  3. Identify the disbursement receipts for the selected health post. From each such receipt record the quantity of drugs which were disbursed to the health post in **Table 10.6,** which lists down the commonly used drugs at the health post. Carefully match the description in the table with the entry in the receipt. If the name of the disbursed drug does not match the list in the table, use the blank rows at the bottom to record the exact name and quantity disbursed.
  4. The blank table should be used to record any spillovers from the main table

| 10.4 | Have you distributed/provided drugs and supplies for health posts under the catchment of this facility during the F.Y. 2006?  ***Yes = 01 No = 02*** | \|  \|  \| \| --- \| --- \|   If “02”, go to the next section (section 11) |
| --- | --- | --- | --- | --- |
| 10.5 | For how many health posts did you distribute drugs and supplies during the F.Y. 2006? | \|  \|  \| \| --- \| --- \| |
| 10.5a | Name of the selected health post | _____________________________ |

**10.6 Drugs distributed to health posts by the health center (MAIN TABLE)**

| **S. No.** | **Product description** | **Unit of issue** | **1** | **2** | **3** | **4** | **5** | **6** | **7** | **8** | **9** | **10** | **11** | **12** |
| --- | --- | --- | --- | --- | --- | --- | --- | --- | --- | --- | --- | --- | --- | --- |
| 1 | Albendazole 400mg, table | Tablet |  |  |  |  |  |  |  |  |  |  |  |  |
| 2 | Amoxacillin 125mg/5ml, syrup | Bottle |  |  |  |  |  |  |  |  |  |  |  |  |
| 3 | Amoxacillin 250mg/5ml, syrup | Bottle |  |  |  |  |  |  |  |  |  |  |  |  |
| 4 | Artesunate Suppository | Suppository |  |  |  |  |  |  |  |  |  |  |  |  |
| 5 | BP 100 Biscuit | Each |  |  |  |  |  |  |  |  |  |  |  |  |
| 6 | Chloroquine 150mg, tablet | Tablet |  |  |  |  |  |  |  |  |  |  |  |  |
| 7 | Chloroquine 150mg/5ml, syrup | Bottle |  |  |  |  |  |  |  |  |  |  |  |  |
| 8 | Coartem 1X6, tablet | Blister |  |  |  |  |  |  |  |  |  |  |  |  |
| 9 | Coartem 2X6, tablet | Blister |  |  |  |  |  |  |  |  |  |  |  |  |
| 10 | Coartem 3X6, tablet | Blister |  |  |  |  |  |  |  |  |  |  |  |  |
| 11 | Coartem 4X6, tablet | Blister |  |  |  |  |  |  |  |  |  |  |  |  |
| 12 | Cotrimoxazole 120mg, tablet | Tablet |  |  |  |  |  |  |  |  |  |  |  |  |
| 13 | Cotrimoxazole 240mg/5ml, syrup | Bottle |  |  |  |  |  |  |  |  |  |  |  |  |
| 14 | Ferrous Sulphate, tablet | Tablet |  |  |  |  |  |  |  |  |  |  |  |  |
| 15 | Folic Acid, tablet | Tablet |  |  |  |  |  |  |  |  |  |  |  |  |
| 16 | Gentian Violet 0.5% | Bottle |  |  |  |  |  |  |  |  |  |  |  |  |
| 17 | Mebendazole 100mg, tablet | Tablet |  |  |  |  |  |  |  |  |  |  |  |  |
| 18 | Mebendazole 500mg, tablet | Tablet |  |  |  |  |  |  |  |  |  |  |  |  |
| 19 | Mebendazole Oral suspn. 100mg/5ml | Bottle |  |  |  |  |  |  |  |  |  |  |  |  |
| 20 | Oral Rehydration Salt | Sachet |  |  |  |  |  |  |  |  |  |  |  |  |
| 21 | Paracetamol 100mg, tablet | Tablet |  |  |  |  |  |  |  |  |  |  |  |  |
| 22 | Paracetamol 120mg/5ml, syrup | Bottle |  |  |  |  |  |  |  |  |  |  |  |  |
| 23 | Paracetamol 125mg, suppository | Suppository |  |  |  |  |  |  |  |  |  |  |  |  |
| 24 | Paracetamol 500mg, tablet | Tablet |  |  |  |  |  |  |  |  |  |  |  |  |
| 25 | RUTF (Plumpy Nut) | Sachet |  |  |  |  |  |  |  |  |  |  |  |  |
| 26 | Tetracycline Eye Ointment, 1% | Tube |  |  |  |  |  |  |  |  |  |  |  |  |
| 27 | Vitamin A 50,000 Unit, caps | Capsule |  |  |  |  |  |  |  |  |  |  |  |  |
| 28 | Vitamin A 100,000 Unit, caps | Capsule |  |  |  |  |  |  |  |  |  |  |  |  |
| 29 | Vitamin A 200,000 Unit, caps | Capsule |  |  |  |  |  |  |  |  |  |  |  |  |
| 30 | Vitamin K, Injection | Ampoule |  |  |  |  |  |  |  |  |  |  |  |  |
| 31 | Zinc 20mg, tablet | Tablet |  |  |  |  |  |  |  |  |  |  |  |  |
| 32 | Condom (male) | Piece |  |  |  |  |  |  |  |  |  |  |  |  |
| 33 | Depoprovera Injection | Vial |  |  |  |  |  |  |  |  |  |  |  |  |
| 34 | Excluton, cycle | Cycle |  |  |  |  |  |  |  |  |  |  |  |  |
| 35 | Implanon ® | Set |  |  |  |  |  |  |  |  |  |  |  |  |
| 36 | Microgynon ®, cycle | Cycle |  |  |  |  |  |  |  |  |  |  |  |  |
| 37 | BCG, Vaccine | Vial |  |  |  |  |  |  |  |  |  |  |  |  |
| 38 | Measles, Vaccine | Vial |  |  |  |  |  |  |  |  |  |  |  |  |
| 39 | Pentavalent, Vaccine | Vial |  |  |  |  |  |  |  |  |  |  |  |  |
| 40 | Polio, Vaccine | Vial |  |  |  |  |  |  |  |  |  |  |  |  |
| 41 | AD Syringe | Piece |  |  |  |  |  |  |  |  |  |  |  |  |
| 42 | Disposable Syringe, 2 ml | Piece |  |  |  |  |  |  |  |  |  |  |  |  |
| 43 | Disposable Syringe, 5 ml | Piece |  |  |  |  |  |  |  |  |  |  |  |  |
| 44 | Gloves (examination) | Pair |  |  |  |  |  |  |  |  |  |  |  |  |
| 45 | Gloves (surgical) | Pair |  |  |  |  |  |  |  |  |  |  |  |  |
| 46 | KHB | Test |  |  |  |  |  |  |  |  |  |  |  |  |
| 47 | RDT | Test |  |  |  |  |  |  |  |  |  |  |  |  |
|  |  |  |  |  |  |  |  |  |  |  |  |  |  |  |
|  |  |  |  |  |  |  |  |  |  |  |  |  |  |  |
|  |  |  |  |  |  |  |  |  |  |  |  |  |  |  |
|  |  |  |  |  |  |  |  |  |  |  |  |  |  |  |
|  |  |  |  |  |  |  |  |  |  |  |  |  |  |  |
|  |  |  |  |  |  |  |  |  |  |  |  |  |  |  |
|  |  |  |  |  |  |  |  |  |  |  |  |  |  |  |
|  |  |  |  |  |  |  |  |  |  |  |  |  |  |  |
|  |  |  |  |  |  |  |  |  |  |  |  |  |  |  |
|  |  |  |  |  |  |  |  |  |  |  |  |  |  |  |

**10.6 Estimation of drugs distributed to health posts by the health center (BLANK TABLE)**

| **S. No.** | **Product description** | **Unit of issue** | **__** | **__** | **__** | **__** | **__** | **__** | **__** | **__** | **__** | **__** | **__** | **__** |
| --- | --- | --- | --- | --- | --- | --- | --- | --- | --- | --- | --- | --- | --- | --- |
|  |  |  |  |  |  |  |  |  |  |  |  |  |  |  |
|  |  |  |  |  |  |  |  |  |  |  |  |  |  |  |
|  |  |  |  |  |  |  |  |  |  |  |  |  |  |  |
|  |  |  |  |  |  |  |  |  |  |  |  |  |  |  |
|  |  |  |  |  |  |  |  |  |  |  |  |  |  |  |
|  |  |  |  |  |  |  |  |  |  |  |  |  |  |  |
|  |  |  |  |  |  |  |  |  |  |  |  |  |  |  |
|  |  |  |  |  |  |  |  |  |  |  |  |  |  |  |
|  |  |  |  |  |  |  |  |  |  |  |  |  |  |  |
|  |  |  |  |  |  |  |  |  |  |  |  |  |  |  |
|  |  |  |  |  |  |  |  |  |  |  |  |  |  |  |
|  |  |  |  |  |  |  |  |  |  |  |  |  |  |  |
|  |  |  |  |  |  |  |  |  |  |  |  |  |  |  |
|  |  |  |  |  |  |  |  |  |  |  |  |  |  |  |
|  |  |  |  |  |  |  |  |  |  |  |  |  |  |  |
|  |  |  |  |  |  |  |  |  |  |  |  |  |  |  |
|  |  |  |  |  |  |  |  |  |  |  |  |  |  |  |
|  |  |  |  |  |  |  |  |  |  |  |  |  |  |  |
|  |  |  |  |  |  |  |  |  |  |  |  |  |  |  |
|  |  |  |  |  |  |  |  |  |  |  |  |  |  |  |
|  |  |  |  |  |  |  |  |  |  |  |  |  |  |  |
|  |  |  |  |  |  |  |  |  |  |  |  |  |  |  |
|  |  |  |  |  |  |  |  |  |  |  |  |  |  |  |
|  |  |  |  |  |  |  |  |  |  |  |  |  |  |  |
|  |  |  |  |  |  |  |  |  |  |  |  |  |  |  |
|  |  |  |  |  |  |  |  |  |  |  |  |  |  |  |
|  |  |  |  |  |  |  |  |  |  |  |  |  |  |  |
|  |  |  |  |  |  |  |  |  |  |  |  |  |  |  |
|  |  |  |  |  |  |  |  |  |  |  |  |  |  |  |
|  |  |  |  |  |  |  |  |  |  |  |  |  |  |  |
|  |  |  |  |  |  |  |  |  |  |  |  |  |  |  |
|  |  |  |  |  |  |  |  |  |  |  |  |  |  |  |
|  |  |  |  |  |  |  |  |  |  |  |  |  |  |  |
|  |  |  |  |  |  |  |  |  |  |  |  |  |  |  |
|  |  |  |  |  |  |  |  |  |  |  |  |  |  |  |
|  |  |  |  |  |  |  |  |  |  |  |  |  |  |  |
|  |  |  |  |  |  |  |  |  |  |  |  |  |  |  |
|  |  |  |  |  |  |  |  |  |  |  |  |  |  |  |
|  |  |  |  |  |  |  |  |  |  |  |  |  |  |  |
|  |  |  |  |  |  |  |  |  |  |  |  |  |  |  |
|  |  |  |  |  |  |  |  |  |  |  |  |  |  |  |
|  |  |  |  |  |  |  |  |  |  |  |  |  |  |  |
|  |  |  |  |  |  |  |  |  |  |  |  |  |  |  |
|  |  |  |  |  |  |  |  |  |  |  |  |  |  |  |
|  |  |  |  |  |  |  |  |  |  |  |  |  |  |  |
|  |  |  |  |  |  |  |  |  |  |  |  |  |  |  |
|  |  |  |  |  |  |  |  |  |  |  |  |  |  |  |
|  |  |  |  |  |  |  |  |  |  |  |  |  |  |  |
|  |  |  |  |  |  |  |  |  |  |  |  |  |  |  |
|  |  |  |  |  |  |  |  |  |  |  |  |  |  |  |
|  |  |  |  |  |  |  |  |  |  |  |  |  |  |  |
|  |  |  |  |  |  |  |  |  |  |  |  |  |  |  |
|  |  |  |  |  |  |  |  |  |  |  |  |  |  |  |
|  |  |  |  |  |  |  |  |  |  |  |  |  |  |  |
|  |  |  |  |  |  |  |  |  |  |  |  |  |  |  |
|  |  |  |  |  |  |  |  |  |  |  |  |  |  |  |
|  |  |  |  |  |  |  |  |  |  |  |  |  |  |  |
|  |  |  |  |  |  |  |  |  |  |  |  |  |  |  |
|  |  |  |  |  |  |  |  |  |  |  |  |  |  |  |
|  |  |  |  |  |  |  |  |  |  |  |  |  |  |  |

1. **FACILITY SERVICES PRICE LIST**

| **Instructions:** 1. Collect a copy of all the charters of pricing for procedures and diagnostic tests being done at the health facility.  2. If a copy is not available note down the full information in the format provided below | | | |
| --- | --- | --- | --- |
|  | | | |
| **S. No.** | **Procedure** | **Price** |  |
|  |  |  |  |
|  |  |  |  |
|  |  |  |  |
|  |  |  |  |
|  |  |  |  |
|  |  |  |  |
|  |  |  |  |
|  |  |  |  |
|  |  |  |  |
|  |  |  |  |
|  |  |  |  |
|  |  |  |  |
|  |  |  |  |
|  |  |  |  |
|  |  |  |  |
|  |  |  |  |
|  |  |  |  |
|  |  |  |  |
|  |  |  |  |
|  |  |  |  |
|  |  |  |  |
|  |  |  |  |
|  |  |  |  |
|  |  |  |  |
|  |  |  |  |
|  |  |  |  |
|  |  |  |  |
|  |  |  |  |
|  |  |  |  |
|  |  |  |  |
|  |  |  |  |
|  |  |  |  |
|  |  |  |  |
|  |  |  |  |
|  |  |  |  |
|  |  |  |  |

1. **Key Informant Interview Guide**

## Instruction

The FMOH in collaboration with Harvard School of Public Health (HSPH) is implementing Resource Tracking and Management (RTM) project. One component of the Resource Tracking and Management (RTM) project in Ethiopia focuses on improving understanding of variability of resource utilization and productivity in primary care services and helping to identify measures that could be employed to improve it as a means of improving health services performance. This questionnaire is prepared to assess and capture relevant data with this regard.

## Respondent Information

- 1. Region _____________________________
  2. Zone _____________________________
  3. Woreda _____________________________
  4. Respondent is from:
     1. Woreda Health Office
     2. Health Facility

If Health Facility

- 1. Facility Type _____________________________
  2. Name of Facility _____________________________

## Interview Information

- 1. Date of Interview _________________
  2. Name of interviewer _____________________________
  3. Name and contact address of the main respondents

| No | Name | Responsibility | Contact details |
| --- | --- | --- | --- |
|  |  |  |  |
|  |  |  |  |
|  |  |  |  |
|  |  |  |  |

# Checklist for KII

**Note: You should ask the facility head or top management for this part**

1. Would you share us your best practices and challenges in relation to resource allocations at the facilities level? (probe with regard to)
   1. Human resources
   2. Drugs and supplies
   3. Equipment
   4. Finance
   5. Other
2. Do you feel that you have adequately (optimally) utilized available resources at your Facility (probe with regard to)
   1. Human resource
   2. Equipment
   3. Beds
   4. Medical Supplies
   5. Budget
   6. Other support from partners
3. Are there major actions carried out by the government or other players to address each of the critical resource allocation challenges identified?
4. What solutions do you suggest to overcome non-optimal resource allocations?
5. What type of support should be sought after from country counterparts for the solution?
