# Supplementary material for: Recurrent costs in primary health care in Ethiopia: facility and disease specific unit costs and their components in government primary hospitals and health centers
Source: BMC Health Serv Res. 2020 May 7;20:389. doi: 10.1186/s12913-020-05218-1 (PMC7204209; doi:10.1186/s12913-020-05218-1)
Supplement: Supplementary file 3 — Additional file 3: Supplementary file 3. Cost accounting steps. This document lays out the specific cost accounting steps used in this costing study. This consists of description of each of the six steps taken: 1) Define the final product; 2) Define the cost centers; 3) Identify and allocate direct costs; 4) Identify and allocate indirect costs; 5) Allocate all costs to cost center; and 6) Compute total and average costs for each final cost center. [file 12913_2020_5218_MOESM3_ESM.docx]

## Supplementary file 1. Cost Accounting Steps

Figure 1 Summary of Cost Accounting Steps

###

### **1. Defining the Final Product**

The first step is defining the final product or services for which unit costs are computed. This depends on several factors, including the purpose of the cost analysis, data availability as well as service provision arrangement. While taking into account these factors we aggregated the outputs to a single index that corresponds well to particular departments for both primary hospitals and health centers. These departments are referred to below with the subscript (k) where k=1….n. For each of these units, the index of outputs is represented by the letter (Q) where q=1…n. Based on this, the output is aggregated to the following indices:

Outpatient (OPD) visits

Inpatient (IPD) discharges

Maternal and Child Health (MCH) visits

Deliveries

### **2. Defining Cost Centers**

The costing analysis is at the level of facility cost centers, which has been defined in such a way that they correspond with the health facilities’ organizational, administrative and accounting structure. Based on this, we have organized all costs into the following three cost centers:

- Patient care cost centers, principally service delivery departments
- Intermediate cost center (pharmacy, laboratory, and diagnostics), and
- Overhead cost centers

### **3. Identify and Allocate Direct Costs**

All relevant inputs (direct and indirect recurrent costs) are identified and assigned to a cost center. First, direct cost are identified and assigned into each cost center. Health post estimates do not include indirect costs.

### **4. Identify and Allocate Indirect Costs**

Once direct costs are allocated to each cost center, the costs assigned to overhead and intermediate cost centers are allocated to patient service cost centers (facility departments) in such a way that it resembles the actual use of resources by each of the department as closely as possible, using the step-down accounting approach. Direct cost of departments providing patient services are used as main allocation factors for indirect cost as appropriate.

### **5. Allocate all costs to final cost center**

Cost estimates are generated only at the level of final cost centers. This is because data on the number of clients served is not available in standardized and uniform formats for intermediate cost centers.

### **6. Compute Total and Average Costs**

For computing unit cost for each cost center, the total cost of each final cost center is divided by the total output of that cost center. Using this approach, at each facility (*i*) we estimate the total costs for each final cost center (*k*) as the sum of direct cost allocated to the cost center plus indirect cost allocated to cost center (*k*) from overhead and intermediate departments

$\boldsymbol{TC}_{\boldsymbol{i,k}}$ **=** $\boldsymbol{DC}_{\boldsymbol{i,k}}$ **+ IC**

The average cost of an index output Q_k_ in unit (*k*) at facility (*i*) is then:

$$\boldsymbol{AC}_{\boldsymbol{k,i}}\boldsymbol{=}\frac{\boldsymbol{TC}_{\boldsymbol{k, i}}}{\boldsymbol{TQ}_{\boldsymbol{k,i}}}$$

By comparing these average costs across the different facility types sampled we have estimated the mean and median costs of specific index outputs (but not specific interventions).
